# Rumen Mycobiome Thiamine Metabolism Contributes to Subacute Rumen Acidosis Tolerance in Goats Through Enhancing Epithelial Cell Proliferation via IGFBP2/IGF1 Axis Activation

**DOI:** 10.1002/exp2.70142

**Published:** 2026-02-24

**Authors:** Jingyi Xu, Xiaodong Chen, Yi Ma, Jianrong Ren, Guanghao Xu, Peilin Zhang, Wenhao Jia, Quanqiu Deng, Yuquan Xiao, Junhu Yao, Shengru Wu

**Affiliations:** ^1^ College of Animal Science and Technology Northwest A&F University Yangling Shaanxi China; ^2^ Key Laboratory of Livestock Biology Northwest A&F University Yangling Shaanxi China; ^3^ College of Animal Science and Technology Ningxia University Yinchuan China; ^4^ School of Life Sciences Jiangsu University Zhenjiang China; ^5^ Laboratory of Metabolic Manipulation of Herbivorous Animal Nutrition College of Animal Science and Technology Yangzhou University Yangzhou China

**Keywords:** miRNA, rumen mycobiome, single‐nucleus transcriptome, subacute rumen acidosis

## Abstract

Subacute rumen acidosis (SARA) is a critical metabolic disorder in dairy ruminants that threatens their milk production. However, the mechanisms by which rumen fungi influence the susceptibility and tolerance of ruminants to SARA remain unclear. This study investigated the relationship between rumen fungal metabolic functions and host SARA tolerance by examining the rumen epithelial miRNA‐mRNA correlations and further cell subtype variations affected by fungi. SARA‐susceptible and SARA‐tolerant goats were identified by monitoring the dynamic rumen pH during high‐concentrate feeding. The abundance of anaerobic fungi and their cellulose and amino acid metabolic functions were reduced in SARA‐susceptible goats, but the enrichment of *Aspergillus bombycis*, which is associated with increased thiamine metabolism, offers potential for SARA tolerance. Furthermore, this study revealed that *Aspergillus bombycis* negatively regulates the rumen epithelial chi‐miR‐17‐26080 through enhanced thiamine metabolism, thereby reducing its inhibitory effect on insulin‐like growth factor binding protein 2 (IGFBP2). The tolerance mechanism of SARA was further explored by adding thiamine to high concentrations, which revealed that the upregulation of IGFBP2 promoted rumen epithelial IGF1 recruitment and increased the content of IGF1, which activated the ERK and PI3K/AKT pathways, further increasing the expression of CDK4 and Cyclin A2 and promoting the rumen epithelial cells' proliferation. The increased number of rumen spinous and basal cells in SARA‐tolerant goats enhances volatile fatty acids absorption in dairy goats, leading to improved SARA tolerance. These findings highlight the roles of rumen fungi and their produced thiamine in preventing SARA in ruminants by enhancing epithelial cell proliferation via the IGFBP2/IGF1 axis activation.

## Graphical abstract

**Schematic illustration of the mechanism underlying the differences between SARA‐susceptible and SARA‐tolerant dairy goats fed a high‐concentrate diet**.

SARA‐susceptible goats (HCS) exhibit low rumen pH and metabolic decline, characterized by epithelial inflammation. Analysis revealed a reduction in common anaerobic fungi, such as *Orpinomyces* and *Piromyces*, along with a decrease in carbohydrate‐degrading enzymes, particularly glycoside hydrolases and carbohydrate‐binding modules. In the rumen epithelium, reduced absorptive epithelial cells lead to decreased volatile fatty acids absorption. Additionally, an increased presence of CD4+ T cells promotes the production of pro‐inflammatory cytokines, further exacerbating inflammation. In comparison, SARA‐tolerant goats maintain a stable rumen pH and normal metabolic function, especially in terms of cellulose degradation. These goats presented a higher abundance of *Aspergillus*, contributing to increased thiamine production. In the rumen epithelium, increased proliferation of spinous and basal cells results in increased VFAs absorption. MicroRNA regulation plays a key role, where chi‐miR‐17‐26080 targets insulin‐like growth factor binding protein 2 and inhibits its translation. A decrease in chi‐miR‐17‐26080 leads to upregulated IGFBP2 expression, which recruits insulin‐like growth factor 1 to the rumen epithelium, activating the IGF1 receptor and subsequently the ERK and PI3K/AKT pathways and promoting epithelial cell proliferation.

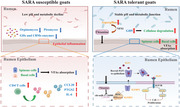

## 1 Introduction

Subacute rumen acidosis (SARA) is a common metabolic disease of dairy cows and goats [1, 2, 3] characterized by a low ruminal pH below 5.6 or 5.8 and lasts for more than 180 min/d [4]. When ruminants develop SARA, the lower ruminal pH, which is caused mainly by the accumulation of volatile fatty acids (VFAs) produced from the microbial fermentation of high‐concentrate diets, can destroy the ruminal epithelial barrier and induce epithelial inflammation [5]. Currently, the difference in the susceptibility of individual dairy ruminants to SARA under high‐concentration diets and its underlying microbial mechanisms have attracted increasing attention [3, 6, 7]. Past research has linked changes in the ruminal microbiome and its metabolites to differences in individualized susceptibility to SARA under a high‐concentrate diet [6]. Nevertheless, the role of microbial components other than rumen bacteria, such as fungi, is largely unexplored in SARA, partly because of their relatively lower abundance and lack of well‐characterized reference genomes [8, 9]. The increased capacity of high‐throughput metagenome sequencing methods has provided access to nonbacterial components of the ruminal microbiota. Although the fungal composition of the ruminal microbiota is estimated to comprise less than 8% of all commensal microbial species by genomic equivalence [10], emerging studies have demonstrated its importance in the rumen [11, 12]. In addition to being pioneers in the degradation of fiber‐containing feed, fungi play crucial roles in regulating nutritional digestion, production performance, and disease prevention and control in ruminants. Among them, *Aspergillus oryzae*, *Aspergillus niger*, and their cultures have been proven to increase fiber degradation capacity, optimize rumen fermentation, and increase production performance [13, 14, 15, 16]. Studies related to human gastrointestinal diseases have shown that fungi and their secondary metabolites, such as acyclic sesquiterpenoids, can alleviate colitis [17]. However, research on how these fungi participate in regulating the digestive and absorptive processes in ruminants through their metabolic functions, as well as the specific metabolic pathways or metabolites involved, remains limited. Thus, an investigation into the ruminal mycobiome in goats with different susceptibilities to SARA would provide a greater understanding of yet unknown mechanisms by which the ruminal microbiome contributes to SARA beyond bacteria. Also, our research would provide a scientific reference for the subsequent regulation of gastrointestinal fungi and metabolism‐related diseases in ruminants.

The accumulation of ruminal VFAs, which is determined by the production of microbial VFAs and the rumen epithelial absorption of VFAs, is the main inducer of SARA. The stratified squamous rumen epithelium is an important region for the rumen absorption and metabolism of VFAs, which can also help maintain a stable ruminal pH and prevent the occurrence of SARA [18]. Furthermore, the rumen epithelium is composed of living strata (stratum basale, spinosum, and granulosum) and corneum (dead cornified keratinocytes) [19], and different rumen cell types perform distinct functions. For instance, studies have shown that ruminal basal and spinous cells uniquely overexpress VFA absorption‐related genes, including solute carrier family 16 member 1 (SLC16A1) and solute carrier family 4 Member 9 (SLC4A9) [18]. Furthermore, the rumen epithelium is an important barrier that performs immune and secretory functions (such as secretory lysozyme and antimicrobial peptides) to protect against pathogenic bacterial infection and is also involved in inflammation during the development of SARA [20, 21]. Hence, we speculated that alterations in ruminal epithelial cell subtypes can regulate differences in the susceptibility of individual dairy ruminants.

Crosstalk between the microbiome and the expression of crucial genes in rumen epithelial cell subtypes was identified in sheep and goat development models [22]. An investigation of the crosstalk between ruminal microbiome alterations and rumen epithelial cell subtypes and their gene expression changes can increase our knowledge of the occurrence of SARA tolerance. Furthermore, microbial regulation of host gene expression can occur through the modulation of differential miRNA expression [23, 24, 25]. miRNAs, comprising 19–25 nucleotides, exert regulatory control over target gene expression by destabilizing target mRNA molecules and inhibiting their translation process [26, 27]. miRNAs play crucial roles in gastrointestinal inflammation by suppressing immune‐ or inflammation‐related gene expression [28]. Furthermore, miRNAs modulate cellular differentiation, proliferation, and apoptosis in response to microbiome alterations through precise regulation of gene expression patterns [29, 30, 31]. Hence, miRNAs are supposed to promote crosstalk between microbial composition and ruminal cell subtypes (and functions) changes that respond to different SARA susceptibilities.

In the present study, we performed single‐nucleus RNA sequencing (snRNA‐seq) on ruminal epithelium to investigate the cell subtypes of dairy goats that contribute to the regulation of SARA susceptibility. Furthermore, ruminal epithelial miRNA sequencing together with ruminal microbiome sequencing was applied to identify potential ruminal microbiome‐miRNA interactions in goats with different SARA susceptibilities. The integration of these data enables the identification of the roles of the differential expression of ruminal miRNAs in mediating the changes in ruminal epithelial cell subtypes that respond to specific microbiome features, especially the mycobiome‐miRNA interaction‐mediated changes in ruminal epithelial cell subtypes, of individual dairy ruminants with differences in SARA susceptibility.

## 2 Results

### 2.1 SARA is Associated With Rumen Epithelial Inflammation in HCS Dairy Goats

**FIGURE 1:**
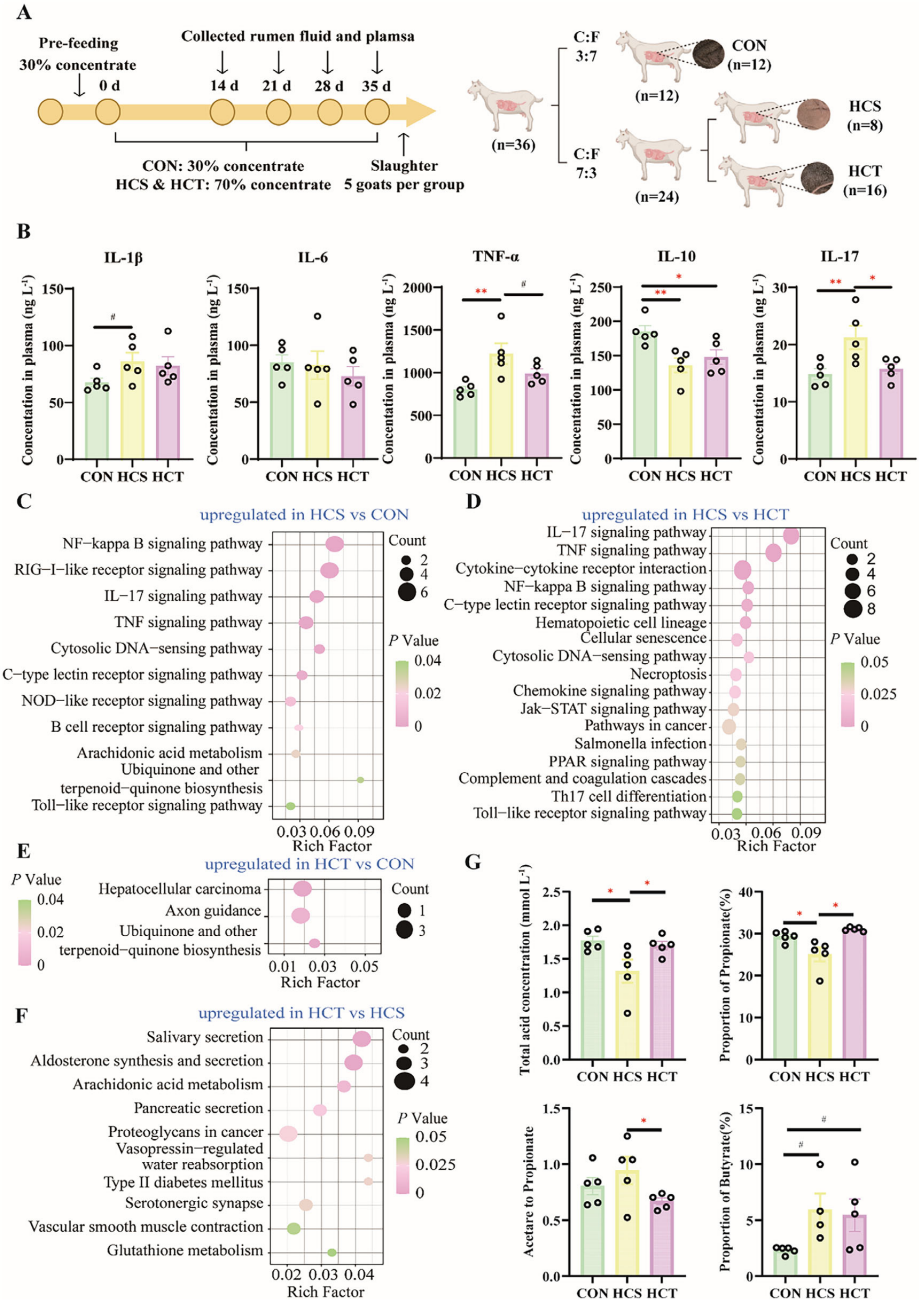
Determination of SARA and epithelial inflammation occurred in HCS goats, whereas VFAs absorption was normal in HCT goats. (A) Twelve dairy goats were randomly selected to feed a low‐concentrate diet at a 3:7 concentrate‐to‐forage ratio as control goats. The remaining 24 dairy goats were selected as the experimental goats by increasing the concentrate to increase the ratio of concentrate to forage to 7:3. Eight goats were identified as SARA goats, and sixteen goats were identified as SARA‐tolerant goats. (B) Comparisons of plasma inflammatory factor levels among CON, HCS, and HCT dairy goats (*n* = 5, separately). (C,D) KEGG enrichment analysis of genes significantly upregulated in HCS dairy goats compared with CON (C) and HCT (D) dairy goats (*n* = 5, separately). (E,F) KEGG enrichment analysis of genes significantly upregulated in HCT dairy goats compared with CON (E) and HCS (F) dairy goats (*n* = 5, separately). (G) The proportion of VFAs in the plasma 6 h after feeding, specifically, the proportions of propionate and butyrate, as well as the ratio of acetate to propionate and the concentration of total VFAs (n = 5, separately). The VFAs' proportions (G) are expressed as the means ± SEM via one‐way ANOVA, followed by the LSD and DUNCAN tests. *p < 0.05, **p < 0.01, ***p < 0.001 indicate significance.

Continuous monitoring of rumen pH dynamic changes for 6 h after afternoon feeding revealed that the pH decreased significantly, and SARA occurred in HCS dairy goats. However, HCT goats did not exhibit SARA occurrence (Figure 1A and Figure S1A, Supporting Information). The determination of plasma inflammatory factors showed that compared to the CON group, the concentration of TNF‐α and IL‐17 increased significantly, whereas the concentration of IL‐10 significantly decreased in the HCS group, and compared to the HCT group, the concentration of IL‐17 of the HCS group also markedly increased (Figure 1B). To explore the holistic gene expression patterns of the rumen epithelium, we conducted epithelial transcriptome sequencing. A total of 11,534 genes were expressed in all three groups: 188 for HCT only, 171 for HCS only, and 151 for CON only (Figure S1B, Supporting Information). KEGG pathway enrichment analysis of the genes upregulated in HCS dairy goats compared with healthy goats (CON and HCT groups) revealed significant enrichment of the NF‒kappa B signaling pathway (*p* < 0.01), the IL‒17 signaling pathway (*p* < 0.01), the TNF signaling pathway (*p* < 0.01), the Toll‒like receptor signaling pathway (*p* < 0.05), the Th17 cell differentiation pathway (*p* < 0.05) and the B‐cell receptor signaling pathway (*p* < 0.05), which are associated with inflammation and immune responses (Figure 1C,D). In the comparison between HCS and CON, as well as between HCS and HCT, the IL−17 signaling pathway, TNF signaling pathway, NF−kappa B signaling pathway, and Toll‐like receptor signaling pathway were significantly enriched simultaneously. The expression of 14 genes involved in these pathways increased in HCS dairy goats, including *EDARADD*, *PTGS2*, *DDX58*, *NFKBIA*, *TNFAIP3*, *RELB*, *CCL20*, *IL6*, *CXCL10*, *MMP3*, *MMP1*, *CASP7*, *EGR3*, and *IRF7* (Figure S1C, Supporting Information). Compared to CON, the genes downregulated in the HCS group were enriched in physiological regulation‐ and signal transmission‐related pathways (Table S4, Supporting Information).

### 2.2 Normal Rumen Epithelial Conditions and VFAs Absorption Capacity Were Maintained in HCT Dairy Goats

KEGG enrichment analysis revealed that the genes upregulated in HCT were significantly enriched in only 3 pathways: hepatocellular carcinoma (*p* < 0.05), axon guidance (*p* < 0.05), and ubiquinone and other terpenoid–quinone biosynthesis (*p* < 0.05; Figure 1E), which are related to cell proliferation, energy metabolism, and molecular and signal transduction. Compared with those in HCS dairy goats, the upregulated genes in HCT dairy goats were significantly enriched in metabolic and humoral secretion pathways, such as salivary secretion (*p* < 0.01) and glutathione metabolism (*p* < 0.05; Figure 1F). The genes enriched in hepatocellular carcinoma that were upregulated in HCT cells, including *WNT2B*, *HMOX1* and *NQO1*, participated in cell proliferation, differentiation, and survival (Figure S2A, Supporting Information). Compared to the CON group, the genes downregulated in the HCT group were enriched in signal transmission‐ and metabolism‐related pathways (Table S4, Supporting Information).

The concentrations of VFAs in the ruminal fluid did not significantly differ, but the concentrations of VFAs in the HCS group were greater than those in the other two groups, whereas the ratio of acetate to propionate was lower than that in the other two groups (Figure S2B, Supporting Information). Unlike the ruminal fluid VFAs, the concentration and proportion of VFAs in the plasma significantly differed. The concentration of total VFAs (*P*_HCS vs CON_ < 0.05, *P*_HCT vs HCS_ < 0.05) and the proportion of propionate (*P*_HCS vs CON_ < 0.05, *P*_HCT vs HCS_ < 0.05) in CON and HCT dairy goats were significantly greater than those in HCS dairy goats. In contrast, the ratio of acetate to propionate (*P*_HCT vs HCS_ < 0.05) in HCT dairy goats was markedly lower than that in HCS dairy goats (Figure 1G). After slaughtering, the number and width of rumen epithelial papillae were detected, and the number of papillae of HCT dairy goats showed an increasing trend compared with that of CON dairy goats (*P*_HCT VS CON < 0.1_, Figure S2C, Supporting Information) Moreover, the width of the papillae on the rumen epithelium in HCT dairy goats was markedly wider than that in CON dairy goats (*P*_HCT vs CON_ < 0.05, Figure S2D, Supporting Information). HE staining of the papillae on the epithelium in the rumen revealed a complete barrier of the stratum corneum and clear boundaries of the cell layers in CON and HCT dairy goats, but shedding of the stratum corneum cells and vague boundaries of the cell layer occurred in HCS dairy goats (Figure S2E, Supporting Information).

### 2.3 Epithelial Cells Increased in HCT Dairy Goats, Whereas CD4^+^ T Cells and Macrophages Increased in HCS Dairy Goats

**FIGURE 2:**
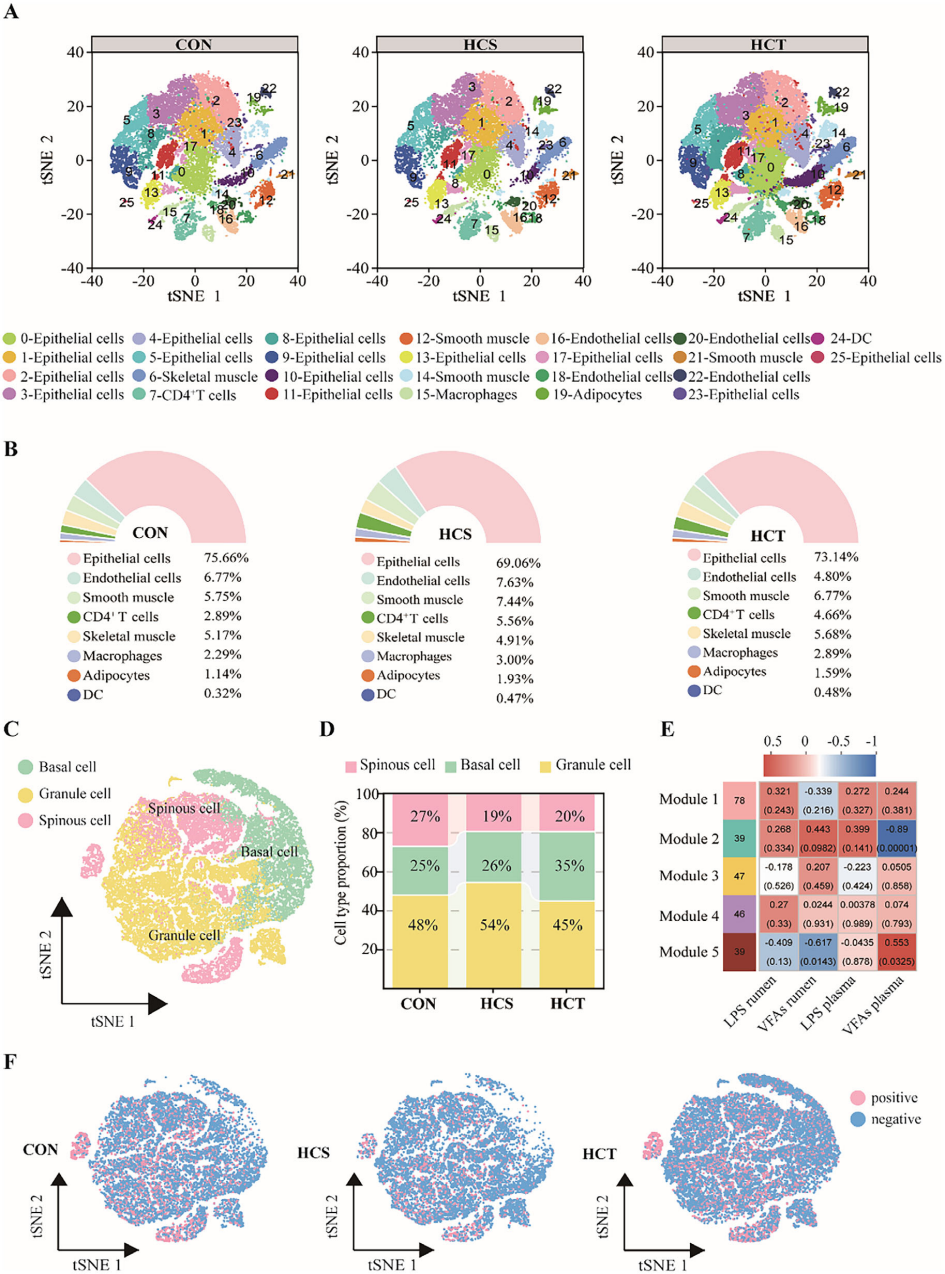
Single‐nucleus RNA transcriptome analysis and cell type identification of the rumen epithelium of CON, HCS, and HCT dairy goats. (A) Distribution of the 26 clusters identified via t‐SNE and the cell types in each cluster in the CON, HCS, and HCT cohorts (*n* = 1, separately). (B) The proportion of different cell types in CON, HCS, and HCT groups (*n* = 1, separately). (C) The distribution of epithelial cell subsets in tSNEs identified by marker genes. (D) The proportions of granule cells, basal cells, and spinous cells in control, HCS, and HCT dairy goats (*n* = 1, separately). (E) Analysis of the transcriptome‐weighted gene co‐expression network between the differentially expressed genes in HCS vs CON, HCT vs CON, and HCT vs HCS, and the phenotypes of the three groups of dairy goats, such as the LPS contents in the rumen and plasma and the VFAs concentrations in the rumen and plasma (*n* = 5, separately). (F) The gene set score shows the degree of activity of the genes related to VFAs absorption in epithelial cell subclusters (*n* = 1, separately).

To investigate the effects of SARA occurrence and the mechanism of SARA tolerance on the rumen epithelium, single‐nucleus RNA sequencing of the rumen epithelium was performed. Using t‐SNE for nonlinear dimensionality reduction, 26 clusters were obtained (Figure 2A). The top three differential expression genes (DEGs) were displayed in Figure S3, Supporting Information. The cell types were noted as epithelial cells (Clusters 0, 1, 2, 3, 4, 5, 8, 9, 10, 11, 13, 17, 23, and 25), endothelial cells (Clusters 16, 18, 20, and 22), CD4^+^ T cells (Cluster 7), macrophages (Cluster 15), adipocytes (Cluster 19), DCs (Cluster 24), skeletal muscle cells (Cluster 6) and smooth muscle cells (Clusters 12, 14, and 21) (Figure 2A and Table S5, Supporting Information). The proportion of cell composition in rumen epithelium of each group was compared, which indicated that the proportion of CD4^+^ T cells and macrophages was greater, whereas the proportion of epithelial cells was lower in HCS dairy goats than in CON and HCT dairy goats (Table S6, Supporting Information, and Figure 2B).

### 2.4 Spinous Cell and Basal Cell Proliferated and VFAs Absorptive Genes Enriched in HCT Dairy Goats

In that case, we performed a second subcluster subdivision of epithelial cells, which were distinguished into 10 clusters (Figure S4A, Supporting Information). The subclusters of epithelial cells were matched with cell markers from the cattle cell landscape at Zhejiang University (Table S7, Supporting Information). Three types of rumen epithelial cells were obtained: spinous cells, basal cells, and granule cells (Figure 2C). Subclusters 2 and 4 were noted as spinous cells, with cell markers such as *CLTB* and *TGM1*. Subclusters 1, 7, and 9 were noted as basal cells, with cell markers such as *ATP5IF1* and *COX5B*. Subclusters 0, 3, 5, 6, and 8 were noted as granule cells with cell markers such as *COL17A1* and *JAG2* (Figure S4B, Supporting Information). Among all the epithelial cell types, granule cells increased and the granular layer thickened, whereas spinous cells and basal cells decreased in HCS dairy goats, indicating that absorptive cells decreased. In contrast, the number of granule cells decreased, but the number of basal cells increased in HCT dairy goats (Table S8, Supporting Information, Figure 2D). To clarify the differential gene distribution in the rumen epithelium, gene set scoring (GSS) was conducted. Compared with those in CON and HCS dairy goats, the expression of genes enriched in spinous cells and basal cells clearly increased in the epithelial cells of HCT dairy goats (Figure S4C, Supporting Information). The DEGs among the HCT, HCS, and CON dairy goats were subjected to weighted gene coexpression network analysis. Five modules were identified: 78 genes for Module 1, 39 genes for Module 2, 47 genes for Module 3, 46 genes for Module 4, and 39 genes for Module 5. The genes in Module 5 were significantly negatively associated with the concentration of rumen VFAs (*p* = 0.0143) and positively associated with the concentration of plasma VFAs (*p* = 0.0325; Figure 2E), which suggested that these genes were related to the absorption of VFAs. These genes were also subjected to GSS, which revealed a well‐defined distribution in spinous cells. In detail, these genes were identified at lower levels in HCS dairy goats than in CON and HCT dairy goats and were more positively expressed in spinous cells of HCT dairy goats (Figure 2F).

### 2.5 Differential Species of Rumen Fungi Occurred Among CON, HCS, and HCT Dairy Goats

**FIGURE 3:**
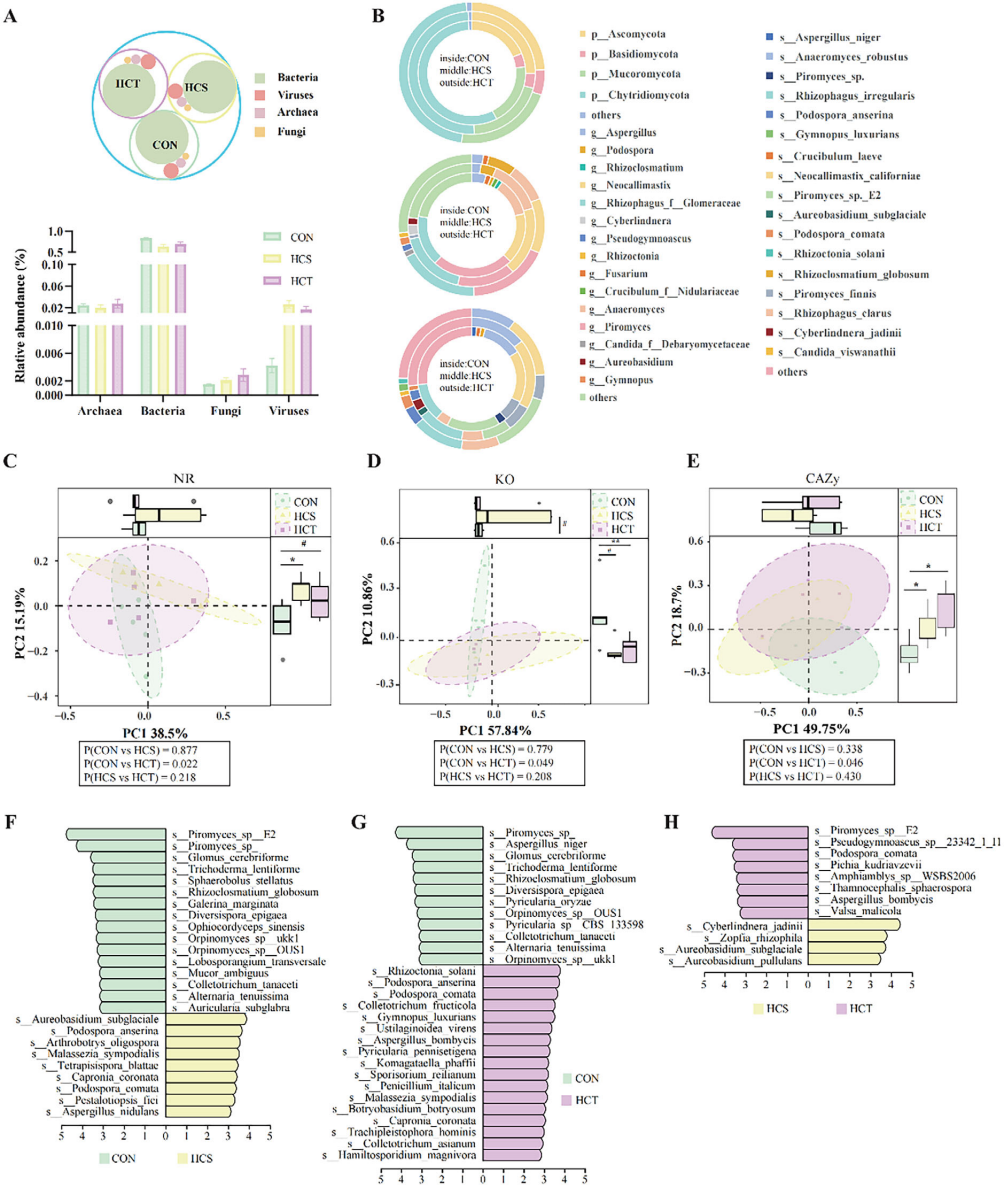
Rumen microbiome differences, especially fungi, among SARA‐susceptible and SARA‐tolerant dairy goats and control goats. (A) Types and quantity of different species of bacteria, viruses, archaea, and fungi among CON, HCS, and HCT dairy goats (*n* = 5, separately). (B) An abundance of fungi in different hierarchies greater than 1% was selected for analysis, and those whose abundance was less than 1% were classified as others in CON, HCS, and HCT dairy goats (*n* = 5, separately). (C–E) The differences in β diversity among CON, HCS, and HCT dairy goats in terms of species (C), KO (D), and CAZy in family level (E) were determined via principal coordinate analysis (PCoA) and a 999 permutations test to determine the significance (*n* = 5, separately). (F–H) LEfSe analysis to screen the different species between HCS and CON (F), between HCT and CON (G), and between HCT and HCS (H), with LDA > 2 and *p* value < 0.05 (*n* = 5, separately).

A statistical analysis of the types and counts of the microbiome in the rumen revealed that 554 species of fungi accounted for 1.71% of the microbiome (Table S9, Supporting Information). A total account of the microbiome revealed that the relative abundance of fungi in HCT dairy goats was greater than that in HCS and CON dairy goats (Table S10, Supporting Information, Figure 3A). Among the fungi with abundances greater than 1%, the four phyla were Chytridiomycota, Mucoromycota, Ascomycota, and Basidiomycota, whereas the top 5 genera were *Piromyces*, *Rhizophagus‐f‐Glomeraceae*, *Neocallimastix*, *Anaeromyces*, and *Aspergillus*. The top 5 species were *Anaeromyces robustus*, *Neocallimastix californiae*, *Piromyces* sp*. E2*, *Rhizophagus_clarus*, and *Rhizophagus irregularis* (Figure 3B). There were no significant differences in the α‐diversities of either the microbiome or fungi among the 3 groups (Figure S5A,B, Supporting Information). Principal coordinate analysis (PCoA) revealed the diversity of the microbiome among the 3 groups of dairy goats, which suggested that the microbiome of high‐concentrate‐fed dairy goats significantly differed from that of CON dairy goats, but there was no marked difference between HCS and HCT dairy goats (*P*_HCS vs CON_ = 0.013, *P*_HCT vs CON_ = 0.009, *P*_HCT vs HCS_ = 0.371; Figure S5C, Supporting Information). However, the PCoA of the rumen fungi in the three dairy goat groups revealed significant differences only between the HCT and CON dairy goats and no notable differences between the HCT and HCS goats or between the HCS and CON goats (*P*_HCS vs CON_ = 0.877, *P*_HCT vs CON_ = 0.022, *P*_HCT vs HCS_ = 0.218; Figure 3C). Moreover, PCoA of rumen fungal KO and CAZy at the family level was also conducted, which revealed that significant differences only occurred between the HCT and CON dairy goats (Figure 3D,E). LEfSe analysis revealed significantly different species between HCS and CON, between HCT and CON, and between HCT and HCS. Compared with those in CON dairy goats, the abundances of 16 species decreased, and those of 9 species increased in HCS dairy goats, whereas the abundances of 12 species decreased and those of 17 species increased in HCT dairy goats. The abundances of typical anaerobic fungi in rumen, like *Orpinomyces* sp *ukk1* (LDA_CON vs HCS_ = 3.22, LDA_CON vs HCT_ = 3.00), *Orpinomyces* sp *OUS1* (LDA_CON vs HCS_ = 3.05, LDA_CON vs HCT_ = 3.11), and *Piromyces* sp (LDA_CON vs HCS_ = 4.17, LDA_CON vs HCT_ = 4.16) were all decreased in HCS and HCT dairy goats (Figure 3F,G). *Podospora comata* (LDA_HCT vs CON_ = 3.56, LDA_HCT vs HCS_ = 3.47) and *Aspergillus bombycis* (LDA_HCT vs CON_ = 3.20, LDA_HCT vs HCS_ = 3.25) both significantly increased in HCT dairy goats compared with HCS and CON dairy goats (Figure 3G,H). Furthermore, the abundance of *Piromyces* sp. *E2* (LDA_CON vs HCS_ = 4.66, LDA_HCT vs HCS_ = 4.48) and *Podospora comata* (LDA_HCS vs CON_ = 3.28, LDA_HCT vs HCS_ = 3.47) were lower in HCS dairy goats than in HCT and CON dairy goats (Figure 3F,H).

### 2.6 Rumen Fungal Specific Functions Helped to Modulate the Occurrence and Tolerance of SARA

**FIGURE 4:**
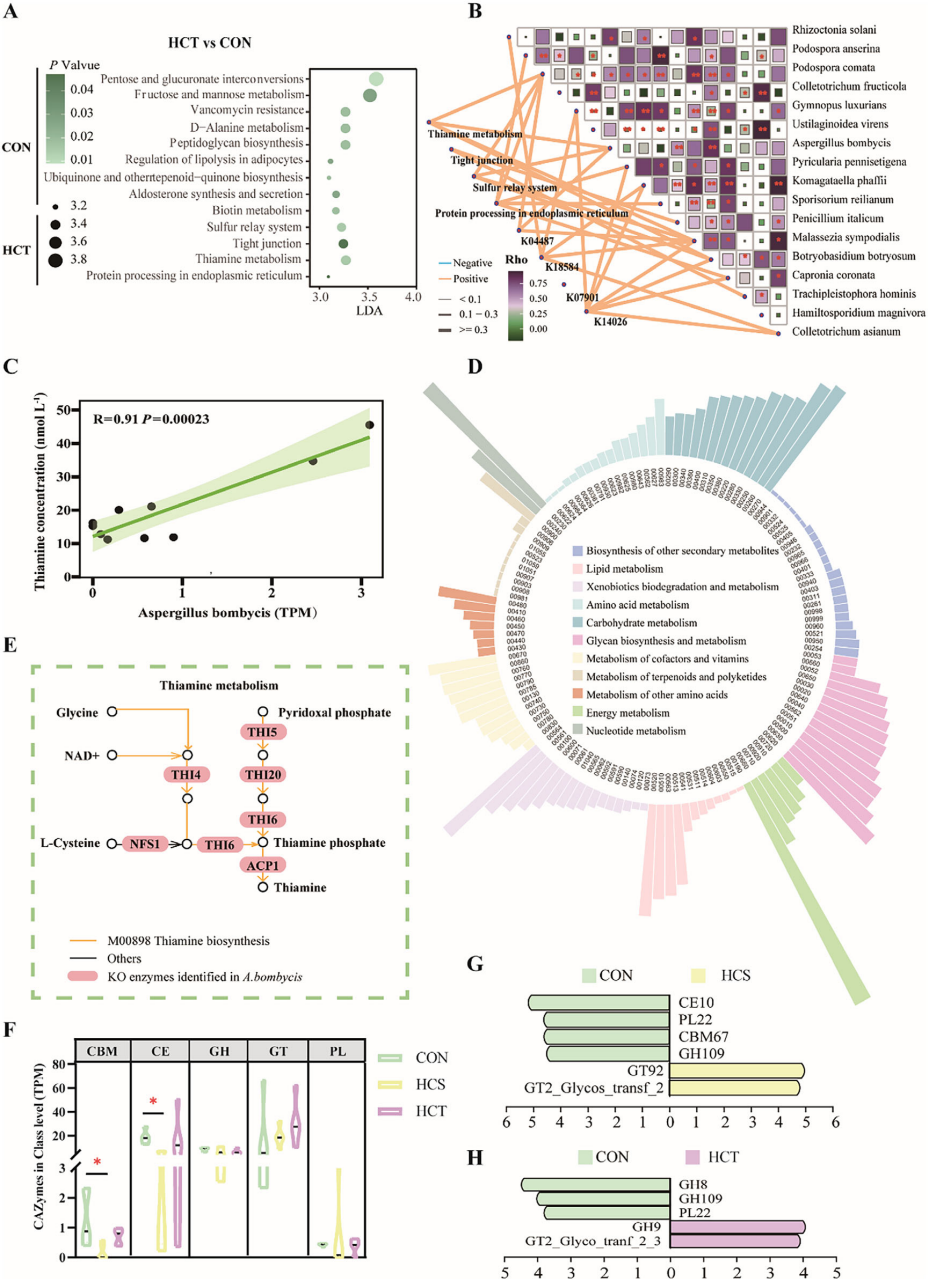
Rumen fungal thiamine metabolism was significantly enhanced in HCT goats, and CAZymes changed among CON, HCS, and HCT dairy goats. (A) LEfSe analysis to screen the significantly differential KEGG pathways at level 3 between HCT and CON groups (*n* = 5, separately). (B) Mantel test between selected KEGG pathways, KOs, and differential species between HCT and CON, which increased in HCT dairy goats, and Spearman correlation analysis revealed the association among fungi species that increased in HCT compared with that in CON dairy goats (*n* = 5, separately). (C) Spearman correlation analysis between the content of thiamine in plasma and the number of TPM of *Aspergillus bombycis*. (D) The KEGG enrichment analysis of *Aspergillus bombycis* in metabolism pathways. (E) The KOs aligned in the *Aspergillus bombycis* genome enriched in thiamine metabolism. (F) Differences in the TPM of CAZymes among CON, HCS, and HCT dairy goats, including 5 modules: glycoside hydrolases (GHs), glycosyltransferases (GTs), polysaccharide lyases (PLs), carbohydrate esterases (CEs), and carbohydrate‐binding modules (CBMs) (*n* = 5, separately). (G,H) LEfSe analysis to screen the significantly differential CAZymes between HCS and CON (G), between HCT and CON (H), with LDA > 2 and *p* value < 0.05 (*n* = 5, separately).

KEGG enrichment analysis revealed that carbohydrate metabolism and amino acid metabolism, such as fructose and mannose metabolism (LDA = 3.78), glycosaminoglycan degradation (LDA = 3.66), galactose metabolism (LDA = 3.51), glycine, serine and threonine metabolism (LDA = 3.73), arginine and proline metabolism (LDA = 3.38) and lysine biosynthesis (LDA = 3.34), significantly decreased in HCS dairy goats compared with CON dairy goats. In contrast, pyrimidine metabolism (LDA = 4.20), purine metabolism (LDA = 4.13), and several disease‐related pathways were significantly enriched in HCS dairy goats (Figure S6A, Supporting Information). However, not similar to HCS goats, thiamine metabolism (LDA = 3.41) and tight junctions (LDA = 3.38) were significantly enriched in HCT dairy goats compared with CON dairy goats (Figure 4A). In addition, oxidative phosphorylation (LDA = 3.41) was significantly enriched in HCS dairy goats compared with that in HCT goats (Figure S6B, Supporting Information).

To clarify whether the fungal metabolic pathways enriched in HCT dairy goats were involved in the regulation of SARA tolerance, LEfSe analysis of KO between HCT and CON was conducted. K04487, named *NFS1* (LDA = 3.41), and K14026, named *SEL1* (LDA = 3.54), were significantly different between HCT and CON dairy goats and participated in significantly different KEGG pathways at the same time (Figure S7A, Supporting Information). Even though the differences of K07901, named *RAB8A*, and K18584, named *ACTR3*, were not significant, but the abundance of these two genes, which participate in tight junctions, was greater in HCT dairy goats (Table S11, Supporting Information). The Mantel test and Spearman correlation analysis revealed associations between significantly different species and fungal functions, which were enriched in HCT dairy goats. *Podospora comata*, *Aspergillus bombycis*, and *Malassezia sympodialis* were strongly positively associated with thiamine metabolism (*R_Podospora comata_* = 0.67, *R _Aspergillus bombycis_* = 0.76, *R_Malassezia sympodialis_* = 0.69) and NFS1 (*R_Podospora comata_* = 0.55, *R_Aspergillus bombycis_* = 0.57, *R_Malassezia sympodialis_* = 0.53). *Podospora comata* was positively correlated with 9 species enriched in HCT dairy goats, including *Aspergillus bombycis* (*R* = 0.56) and *Malassezia sympodialis* (*R* = 0.83; Figure 4B). However, the association analysis between the content of thiamine in the plasma and these three fungi indicated that only *Aspergillus bombycis* was significantly positively related to the content of thiamine (R = 0.91, *p* < 0.001; Figure 4C, Figure S7B,C, Supporting Information).

To further investigate whether *Aspergillus bombycis* has the capability to synthesize thiamine, we downloaded the genome sequence of a single strain for subsequent analysis, with a total genome assembly length of 37,476,653 bp and a GC content of 48.7%. Gene prediction revealed that 12,263 genes were annotated, with 4,755 genes annotated in the KEGG database (Table S12, Supporting Information). To determine the potential roles of *Aspergillus bombycis*, specific KOs that participate in metabolism were analyzed. A total of 145 pathways related to metabolism were enriched. Among them, 109, 94, and 88 genes were annotated in the biosynthesis of cofactors, biosynthesis of amino acids, and carbon metabolism, respectively (Figure 4D). A total of 10 KOs involved in thiamine metabolism were annotated, including *NFS1*, which was previously identified through metagenomic analysis. These findings indicate that *Aspergillus bombycis* possesses the capability to independently carry out de novo thiamine synthesis (Figure 4E).

Compared with those of CON dairy goats, the carbohydrate esterases (CEs) and carbohydrate‐binding modules (CBMs) of HCS dairy goats were significantly lower (Figure 4F). The LEfSe analysis of CAZy revealed that PL22 (LDA_CON vs HCS_ = 3.80, LDA_CON vs HCT_ = 3.11) and GH109 (LDA_CON vs HCS_ = 4.14, LDA_CON vs HCT_ = 3.93) both decreased in high‐concentrate‐fed goats (Figure 4G,H). However, in HCT dairy goats, the cellulose‐degrading enzyme GH9 was increased compared with that in CON dairy goats (LDA = 4.00), and the glycosyl transferase GT2 Glyco tranf 2–3 (LDA = 3.85) was increased compared with that in CON dairy goats (Figure 4G,H).

### 2.7 miRNAs Inhibited Rumen Epithelial Gene Expression to Regulate Rumen Epithelial Cell Proliferation and Metabolism

**FIGURE 5:**
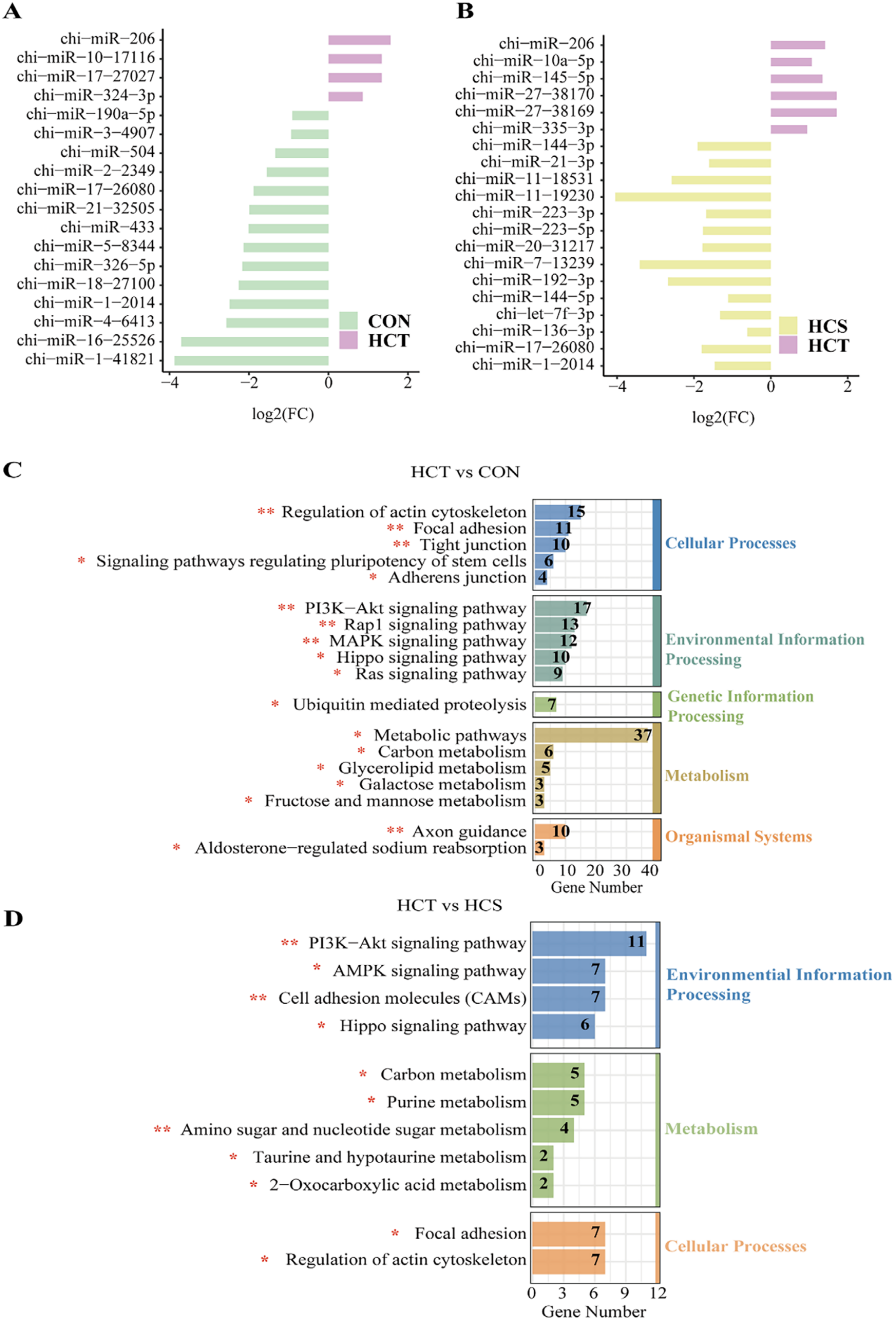
Effects of miRNA regulation of epithelial gene expression on tolerance to SARA and coregulation of host gene expression. (A) The expression of miRNAs in the rumen epithelium of dairy goats was significantly different between the CON and HCT dairy goats (*n* = 5, separately). (B) The expression of miRNAs in the rumen epithelium of dairy goats was significantly different between the HCT and HCS dairy goats (*n* = 5, separately). (C) Compared with those in CON dairy goats, the downregulated miRNAs in HCT dairy goats were analyzed by KEGG enrichment after target gene prediction (*n* = 5, separately). (D) Compared with those in HCS dairy goats, the downregulated miRNAs in HCT dairy goats were analyzed by KEGG enrichment after target gene prediction (*n* = 5, separately).

miRNAs can be used as vectors for the microbiome to regulate host gene expression. We detected the miRNA expression profile of the rumen epithelium. To explore the roles of miRNA in regulating rumen epithelial cell proliferation and the tolerance of SARA, we next focused on differences in the miRNA transcriptome between HCT dairy goats and the other two groups of goats. Compared with those in CON dairy goats, the expression of 4 miRNAs in HCT goats increased significantly, whereas the expression of 14 miRNAs decreased significantly (*p* < 0.05; Figure 5A). Compared with those in HCS goats, the expression of 6 miRNAs was significantly upregulated, and the expression of 14 miRNAs was significantly downregulated in HCT goats (*p* < 0.05; Figure 5B). The target genes of the downregulated miRNAs in HCT goats were predicted, and KEGG pathway enrichment analysis was performed. Compared with those in the CON dairy goats, the target genes of the downregulated miRNAs in the HCT goats were enriched mainly in focal adhesion (*p* < 0.01), tight junction (*p* < 0.01), and regulation of the actin cytoskeleton (*p* < 0.01), which mediate tight arrangement. Additionally, the Rap1 signaling pathway (*p* < 0.01), Hippo signaling pathway (*p* < 0.01), PI3K‐Akt signaling pathway (*p* < 0.01), and MAPK signaling pathway (*p* < 0.05), which are known to mediate cell differentiation, proliferation, and apoptosis, were significantly enriched. Metabolism‐related pathways, such as those related to glycerolipid metabolism (*p* < 0.05), galactose metabolism (*p* < 0.05), carbon metabolism (*p* < 0.05), and fructose and mannose metabolism (*p* < 0.05; Figure 5C and Figure S8A, Supporting Information), were also significantly enriched. Similar to the differences between HCT and CON dairy goats, the target genes of the downregulated miRNAs in the HCT group compared with those in the HCS group were also enriched in pathways such as the PI3K‐Akt signaling pathway (*p* < 0.05), the Hippo signaling pathway (*p* < 0.05), focal adhesion (*p* < 0.05), regulation of the actin cytoskeleton (*p* < 0.05), and carbon metabolism (*p* < 0.05). The remaining DEGs were associated with cell proliferation, for example, the AMPK signaling pathway (*p* < 0.01) and the cell adhesion molecule (CAM) pathway (*p* < 0.01), and were associated with metabolic pathways, such as purine metabolism (*p* < 0.05), amino sugar and nucleotide sugar metabolism (*p* < 0.01) and taurine and hypotaurine metabolism (*p* < 0.05; Figure 5D and Figure S8B, Supporting Information).

### 2.8 Reducing the Inhibitory Effect of miRNAs on mRNAs in HCT Goats Promoted Metabolism, Absorption, and Cell Proliferation

**FIGURE 6:**
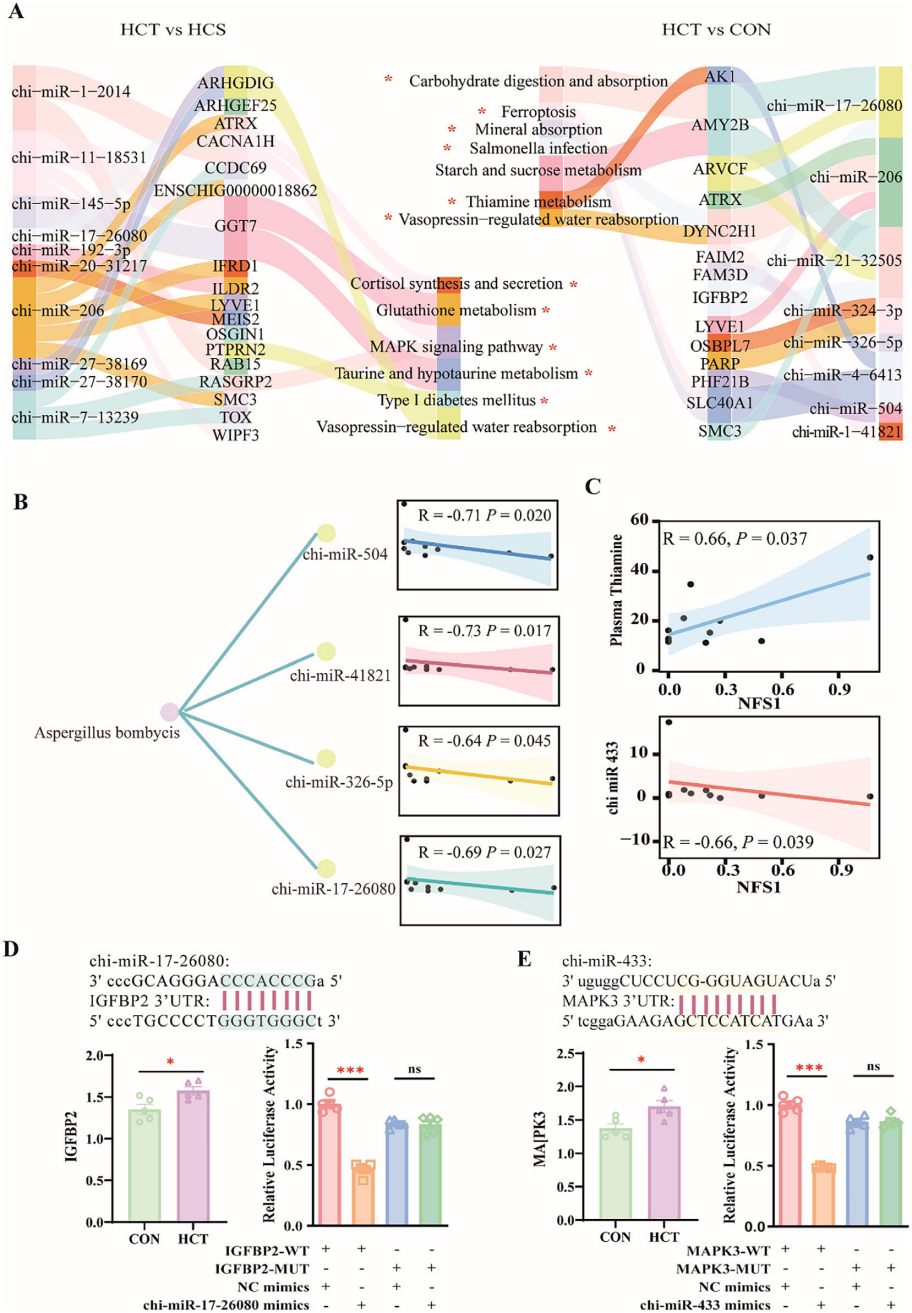
Multi‐omics analysis revealed the mechanism by which rumen fungi affected miRNAs in the rumen epithelium to modulate gene expression, which ultimately affected the number of epithelial cells and VFAs absorption. (A) Negative correlations between miRNAs and mRNAs were screened via miRNA‒mRNA association analysis, among which miRNAs that were downregulated in HCT dairy goats compared with CON and HCS dairy goats and their target genes were screened, and KEGG enrichment analysis was performed (*n* = 5, separately). (B) Spearman correlation analysis between *Aspergillus bombycis* and significantly downregulated miRNAs in HCT dairy goats compared with CON dairy goats (*n* = 10). (C) Spearman correlation analysis between the content of thiamine in plasma and the number of TPM of *NFS1*, as well as between the expression of chi‐miR‐433 and the number of TPM of *NFS1* (*n* = 10). (D) chi‐miR‐17‐26080 targeted IGFBP2, and the relative expression of *IGFBP2* in the rumen epithelium, represented by log_10_(TPM), along with the results of the dual‐luciferase reporter gene assay (*n* = 5, separately). (E) chi‐miR‐433 targeted *MAPK3*, and the relative expression of *MAPK3* in the rumen epithelium, represented by log_10_(TPM), along with the results of the dual‐luciferase reporter gene assay (*n* = 5, separately).

To further explore the regulatory effect of miRNAs on rumen epithelial gene expression, we performed miRNA‒mRNA interaction analysis. A total of 10 differentially expressed miRNAs negatively corresponding to 18 DEGs were detected in both the transcriptome and the miRNA transcriptome of HCT and HCS goats at the same time (*p* < 0.05; Figure 6A). The 18 genes were *ARHGDIG*, *ARHGEF25*, *ATRX*, *CACNA1H*, *CCDC69*, *ENSCHIG00000018862*, *GGT7*, *IFRD1*, *ILDR2*, *LYVE1*, *MEIS2*, *OSGIN1*, *PTPRN2*, *RAB15*, *RASGRP2*, *SMC3*, *TOX*, and *WIPF3*. KEGG enrichment analysis revealed that these genes were enriched in cortisol synthesis and secretion, glutathione metabolism, the MAPK signaling pathway, taurine and hypotaurine metabolism, type 1 diabetes mellitus, and vasopressin‐regulated water reabsorption (*p* < 0.05; Figure 6A). *CACNA1H* and *RASGRP2* are enriched in the MAPK signaling pathway and participate in regulating cell proliferation and differentiation. The miRNA‒mRNA pairs with negative correlations between gene expression and miRNA expression were screened between HCT and CON dairy goats, and a total of 14 genes and 8 miRNAs were identified. The 14 genes were *ARVCF*, *AMY2B*, *ATRX*, *DYNC2HI*, *LYVE1*, *OSBPL7*, *PARP*, *SMC3*, *IGFBP2*, *SLC40A1*, *AK1*, *FAIM2*, *FAM3D*, and *PHF21B*. Seven pathways were enriched: carbohydrate digestion and absorption, ferroptosis, mineral absorption, Salmonella infection, starch and sucrose metabolism, thiamine metabolism, and vasopressin‐regulated water reabsorption (*p* < 0.05; Figure 6A).

### 2.9 Multi‐Omics Association Analysis Elucidates Fungus‐Mediated miRNA Regulation of SARA Tolerance

According to the above analysis results, key fungi *Aspergillus bombycis*, key function of thiamine metabolism, and key enzyme *NFS1* were identified, which elucidated that those fungi made efforts to provide thiamine to the host. However, the mechanism by which fungi regulate host gene expression and function requires further analysis. Correlation analysis revealed that *Aspergillus bombycis* was negatively associated with chi‐miR‐504 (*R* = −0.71, *p* = 0.02), chi‐miR‐41821 (*R* = −0.73, *p* = 0.017), chi‐miR‐326‐5p (*R* = −0.64, *p* = 0.045) and chi‐miR‐17‐26080 (*R* = −0.69, *p* = 0.027) (Figure 6B). An exploration of the relationship between *NFS1* and the content of thiamine revealed that *NFS1* was positively related to the content of plasma thiamine (R = 0.66, *p* = 0.037; Figure 6C). Moreover, chi‐miR‐433 downregulation in HCT dairy goats compared with CON dairy goats was significantly negatively associated with *NFS1* (*R* = −0.66, *p* = 0.039; Figure 6C). These miRNAs negatively associated with *Aspergillus bombycis* were significantly downregulated in HCT compared to CON dairy goats. *IGFBP2*, which was targeted by chi‐miR‐17‐26080, was significantly upregulated in HCT dairy goats (Figure 6D and Figure S9A, Supporting Information). As expected, co‐transfection assay with chi‐miR‐17‐26080 mimics revealed that chi‐miR‐17‐26080 induced downregulation of the reporter vector of *IGFBP2* 3′‐UTR, whereas it had no effect on the reporter with mutated *IGFBP2* 3′‐UTR (*p* < 0.001; Figure 6D). *LEP*, *SGK1*, and *MAPK3*, these genes were also all targeted by chi‐miR‐433 (Figure 6E and S9B, Supporting Information), and participate in immune processes, cell survival, and proliferation. Among them, the relative expression of *MAPK3* significantly increased in HCT goats (*p* < 0.05), and the dual‐luciferase reporter gene system revealed that chi‐miR‐433 inhibited the transcriptional activity of *MAPK3* (*p* < 0.001; Figure 6E).

### 2.10 Dietary Thiamine Supplementation Could Prevent Epithelial Inflammation and Activated the IGFBP2‐IGF1‐ERK and IGFBP2‐IGF1‐AKT Pathways to Promote Cell Proliferation

**FIGURE 7:**
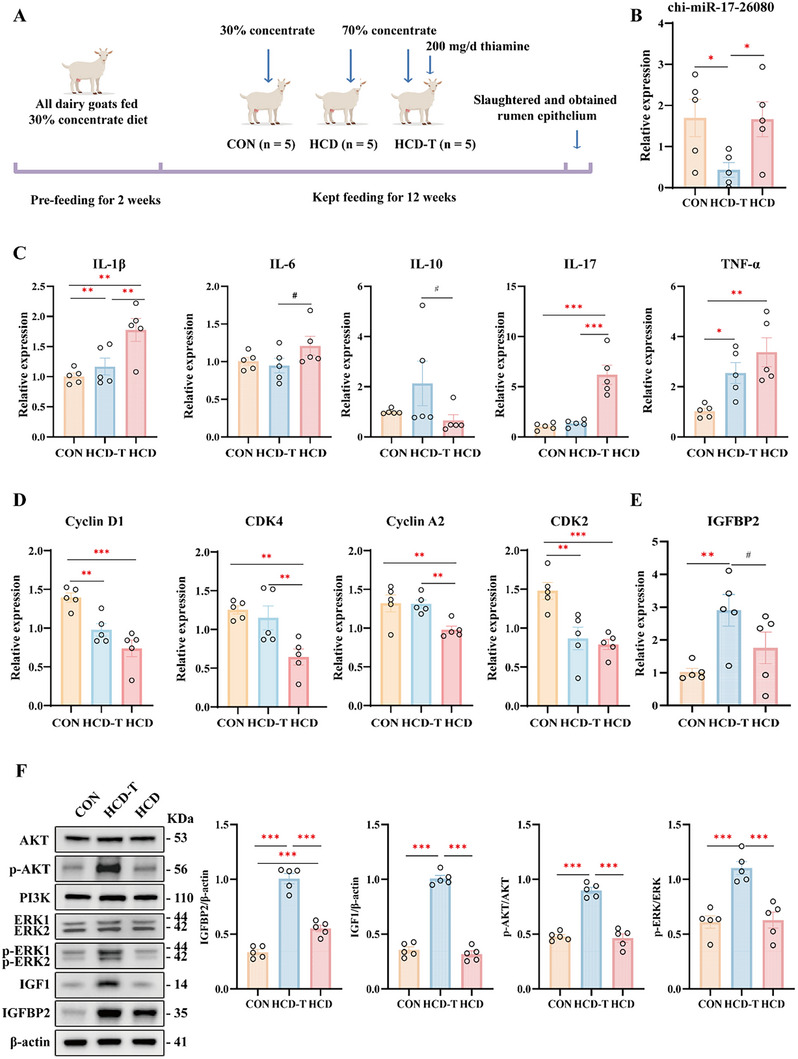
Adding thiamine to a high‐concentrate diet prevented epithelial inflammation and activated the IGFBP2/IGF1‐PI3K/AKT and IGFBP2/IGF1‐ERK signaling pathways to promote cell proliferation. (A) Twenty‐four dairy goats were randomly divided into three groups. The CON group was fed a 30% concentrate diet, whereas the HCD and HCD‐T groups were fed a 70% concentrate diet. Additionally, 200 mg thiamine per kilogram of dry matter was added to the feed of the HCD‐T group. After the feeding trial, five goats from each group were slaughtered, and their rumen epithelium was collected for further analysis (*n* = 5, separately). (B) Relative expression of chi‐miR‐17‐26080 in the rumen epithelium (*n* = 5, separately). (C) Relative expression of inflammation‐related cytokines, including *IL‐1β*, *IL‐6*, *IL‐10*, *IL‐17*, and *TNFα*, in the rumen epithelium (*n* = 5, separately). (D) Relative expression of cell proliferation‐related genes, including *Cyclin D1*, *CDK4*, *Cyclin A2*, and *CDK2*, in the rumen epithelium (*n* = 5, separately). (E) The relative expression of *IGFBP2* in the rumen epithelium (*n* = 5, separately). (F) The protein levels of IGFBP2, IGF1, AKT, PI3K, and ERK1/2 and the phosphorylation of AKT and ERK1/2 (*n* = 5, separately). All the statistical analyzes were performed via one‐way ANOVA, with the results presented as the means ± SEMs, followed by the LSD and DUNCAN tests. *p < 0.05, **p < 0.01, and ***p < 0.001 indicate statistical significance.

To verify the role of thiamine in SARA tolerance and epithelial cell proliferation, 200 mg thiamine per kilogram of dry matter was added to the feed of the HCD‐T group, while the CON group continued to receive a diet containing 30% concentrate. Both HCD and HCD‐T goats were fed diets with 70% concentrate (Figure 7A). The relative expression of chi‐miR‐17‐26080 significantly decreased in HCD‐T goats (*p* < 0.05, Figure 7B). The relative expression of IL‐1β and *IL‐17* in the rumen epithelium of HCD goats was significantly greater than that in the rumen epithelium of CON‐ and HCD‐T goats (*p* < 0.05, Figure 7C). Compared with that in the CON group, the expression of TNFα significantly increased in dairy goats fed a high‐concentrate diet (*p* < 0.05, Figure 7C). Compared with those in HCD‐T goats, there was a decreasing trend in *IL‐10* and an increasing trend in *IL‐6* in HCD goats (Figure 7C). Detection of the expression of genes associated with cell proliferation revealed that the expression levels of *Cyclin D1* and CDK2 were significantly reduced under high‐concentration feeding conditions (*p* < 0.01; Figure 7D). However, in the HCD‐T group, the expression levels of CDK4 and *Cyclin A2* were comparable to those in the CON group and markedly higher than those in the HCD group (*p* < 0.01; Figure 7D). These findings suggested that thiamine supplementation could enhance the proliferation of epithelial cells. Notably, the relative expression of *IGFBP2* was significantly greater in HCD‐T goats than in CON goats, which was consistent with the previous hypothesis (*p* < 0.01, Figure 7e), but there was no significant difference in *IGF1* (Figure S10A, Supporting Information). IGFBP2, as a binding protein of IGF1, can modulate the activity of IGF1 and participate in cell proliferation by stimulating PI3K/AKT or MAPK signaling. To determine the effect of increased IGFBP2 expression, the protein levels of IGFBP2 and IGF1 and the phosphorylation of AKT and ERK were examined. Compared with those in CON and HCD goats, the protein expression of IGFBP2 and IGF1, the phosphorylation of AKT, and the phosphorylation of ERK and PI3K significantly increased in HCD‐T goats (*p* < 0.001, Figure 7F and Figure S10B, Supporting Information).

## 3 Discussion

Accompany with the rise of the human population, the need for milk products has increased rapidly in recent years. Goat milk is gaining popularity because of its unique flavor, high protein and short‐chain fatty acid contents, low milk allergies, and gastrointestinal disorders [32]. Receiving a high‐energy diet is a double‐edged sword, as it can increase the milk production of dairy goats while also increasing the risk for SARA. SARA is one of the most common and severe metabolic diseases affecting milk yield and ruminant health [33, 34]. Different individuals showed discrepant SARA susceptibility and tolerance, but the microbial functions involved are unknown, especially the role of fungi. Our study confirmed that a high‐concentration diet increased the risk of SARA in dairy goats and constructed a model of SARA‐susceptible and SARA‐tolerant dairy goats. This is one of the few studies that systemically analyzed fungal composition and function and metabolite changes in response to a high‐concentration diet. In addition to the host ruminal transcriptome and single‐nucleus transcriptome results, our study identified the roles of fungi and their metabolic functions in both the regulation of SARA occurrence and SARA tolerance. It is worth noting that previous studies have demonstrated that supplementing ruminant diets with fungi such as *Aspergillus oryzae* and *Aspergillus niger* can effectively mitigate the occurrence of metabolic disorders and enhance production performance [14, 15]. However, these studies have largely overlooked the specific mechanisms through which these fungi exert their effects, as well as their roles in modulating host disease occurrence and metabolism. To the best of our knowledge, this study is the first to investigate the regulatory mechanisms underlying SARA tolerance by focusing on the metabolic functions of fungi.

Our studies revealed that the occurrence of SARA led to noticeable pathological changes in the rumen epithelial papillae, affecting the absorption of VFAs by the rumen epithelium, particularly the absorption of propionate. This can decrease the pH of the rumen and affect dairy goats’ absorption and utilization of VFAs, which can serve as a potential mechanism of the harmful effects of SARA on lactation performance. Moreover, compared with previous studies that focused mainly on rumen bacterial composition changes, the present study assessed ruminal fungal composition changes in dairy goats when they exhibited SARA tolerance and susceptibility [35]. In the present study, the 4 previously reported anaerobic fungal species that play crucial roles in the degradation of lignocellulosic plant material in the rumen, including ruminal *Piromyces* sp., *Orpinomyces* sp. UKK1, *Orpinomyces* sp. OUS1, and *Piromyces* sp. *E2* [36, 37, 38, 39] were significantly lower in the SARA goats than in the CON goats. Compared with that in HCS goats, the relative abundance of *Piromyces* sp. *E2* increased in both CON and HCT goats. Hence, the decrease in these anaerobic fungal species can also explain the reduced fiber digestion in the rumen when SARA occurs. In contrast, *Podospora comata* and *Aspergillus bombycis* were significantly greater in the HCT group than in the CON and HCS groups. These two species have not been fully researched, but the genera *Podospora* and *Aspergillus* play important roles in fiber digestion [40, 41, 42, 43]. These results indicated that HCT goats have better fiber digestion abilities than HCS goats do and that these fungi may also contribute to the regulation of SARA tolerance.

Furthermore, previous studies have focused on changes in ruminal bacteriome function in response to high‐concentration diets and varied susceptibilities to SARA [44, 45]. These changes included an enrichment of genes involved in carbohydrate metabolism, amino acid metabolism, and biofilm formation [44, 45]. Briefly, the increase in GH13, CBM26, and CBM34 enzymes in high‐concentration diets of the previous study suggested a shift in the ability of the rumen microbiota to break down starch, which was abundant in these diets. Conversely, the abundance of cellulases (GH5, GH9, and GH51) decreased, indicating a shift away from cellulose degradation [46]. Similarly, our research revealed fewer enzymes of the CE and CBM modules in HCS goats and more cellulases of the GH9 module in HCT goats than in CON goats. This suggested that the overall carbohydrate metabolism capacity in the rumen of HCS dairy goats was reduced, while the carbohydrate metabolism of HCT dairy goats was not greatly affected, but the fiber degradation capacity was increased. Furthermore, high‐concentrate diets led to perturbations in the contents of amino acids and their corresponding metabolic pathways [47, 48]. The abundance of genes involved in amino acid metabolism, such as those encoding enzymes for aminoacyl‐tRNA biosynthesis, was decreased in high‐concentrate diet‐fed animals [49]. This indicated a shift in the metabolic priorities of the rumen microbiota towards energy production rather than protein synthesis. This adaptation may be beneficial for the host in the short term but could have long‐term implications for the balance of amino acids in the rumen and the overall health of the animal [49]. Compared with previous studies that focused mainly on changes in rumen bacterial function, we first assessed changes in ruminal fungal function in dairy goats fed high‐concentration diets. Similar to previous studies focused on bacteriome function changes, the fungi in the SARA dairy goats presented significantly reduced functions in carbohydrate metabolism and amino acid metabolism, suggesting a decrease in metabolic capability within the rumen compared with healthy dairy goats from the CON group. Moreover, in a previous study, SARA was associated with a decrease in antioxidant capacity, and an increase in inflammatory cytokines such as IL‐1β, IL‐4, and IL‐6 can lead to oxidative stress and inflammation, which negatively impact animal performance and welfare [45]. In the present study, compared with those in the SARA‐tolerant dairy goats, the ruminal fungal function in the SARA‐susceptible dairy goats was significantly enriched in infection‐ and antioxidant‐related pathways, suggesting an increased risk of oxidative stress or an inflammatory state within the rumen. Hence, ruminal fungal functions, including carbohydrate and amino acid metabolic functions, were weaker in SARA goats than in healthy goats. However, the carbohydrate metabolic function of goats in the HCT group was more similar to that of CON dairy goats, which indicated a more stable ruminal fungal function in the HCT group.

It has been reported that the variation in the expression of the TLR/MyD88‐NFκB pathway genes in the rumen epithelial wall significantly changed in ruminants with SARA differences in susceptibility and tolerance [50, 51]. Our study further confirmed that the ruminal IL‐17 signaling pathway, which was previously suggested to increase the activation of NF‐κB and other proinflammatory cytokines [52], was increased in SARA goats but inhibited in goats in the HCT group. Unlike the TLR/MyD88‐NFκB‐mediated nonspecific immune response, which may not be easily regulated by the immune system, the IL‐17 signaling pathway can be more easily regulated and can serve as a key to regulating appropriate immunity and preventing excessive inflammation [53, 54]. Hence, our findings led to the speculation that rumen inflammation in SARA goats and the inhibition of rumen inflammation in SARA‐tolerant goats can be initiated through the regulation of the IL‐17 signaling pathway. Moreover, except for the ruminal inflammation differences between goats with SARA susceptibility or tolerance, the ruminal environment of goats from the HCT group, including the pH, LPS content, and VFAs composition, was more similar to that of healthy control goats. Previous studies have implied that the ruminal pH and VFAs composition are determined by the production and absorption of VFAs [55, 56]. Our single‐nucleus transcriptomic and transcriptome analysis revealed significantly more epithelial cells within the SARA‐tolerant groups than in the SARA‐susceptible and healthy control groups. Among these identified epithelial cells, basal cells presented the most prominent increases, with these cell types previously suggested to be responsible for VFA absorption [57]. Hence, we hypothesized that the increased basal cell proliferation in the HCT group promoted ruminal VFAs absorption, helped maintain the stability of the ruminal environment, and prevented the occurrence of SARA.

Notably, increased thiamine metabolism by ruminal fungi, increased fungal thiamine metabolism‐related enzymes, and increased thiamine composition in plasma were identified in the HCT group. In previous studies, dietary thiamine supplementation was shown to reduce apoptosis in rumen epithelial cells, promote cell proliferation, and maintain the normal morphological structure and function of mitochondria [58, 59, 60]. These combined improvements protect rumen epithelial barrier function through supplementation with thiamine [60]. Furthermore, thiamine inhibits the NF‐κB/p38 MAPK/AMPK pathway, reducing cellular inflammation and maintaining normal energy metabolism status, which serves as an important mechanism for the protection of the rumen epithelial barrier by thiamine [61]. In line with these previous studies, the present study revealed that increased *Aspergillus bombycis* may serve as the key fungus contributing to the increased fungal thiamine metabolism in the HCT group and promoting epithelial proliferation in high‐concentrate‐fed goats. A recent study revealed that the genus *Aspergillus* contains all the enzymes needed for thiamine synthesis [17]. Our analysis of the genome of *Aspergillus bombycis* also revealed that it could synthesize thiamine de novo on its own. Hence, we hypothesized that ruminal fungi such as *Aspergillus bombycis*, as well as enhanced fungal thiamine synthesis, can contribute to ruminal basal cell proliferation activation and the inhibition of SARA, which has been proven by the direct feeding of thiamine to high‐concentration diet‐induced SARA goats.

To identify the potential mechanism that mediates the activation of ruminal basal cell proliferation, the ruminal epithelial miRNA profiles were identified. Previous studies have shown that miRNAs can regulate ruminal epithelial cell apoptosis and inflammation processes when SARA occurs [62]. In contrast to these previous studies, our study revealed that the downregulated miRNAs in the HCT group were involved in the regulation of cell proliferation and epithelial barrier homeostasis compared with those in the CON and HCS groups. Furthermore, systemic analysis on the basis of rumen epithelial miRNA profiles together with the epithelial transcriptome and ruminal mycobiome revealed that decreased chi‐miR‐17‐26080 and chi‐miR‐433 can be mediated by the roles of increased fungal thiamine synthesis in promoting *IGFBP2*, *LEP*, *SGK1*, and *MAPK3* expression. A previous study suggested that IGFBP2 can promote the expression of IGF1, activate the ERK and PI3K/AKT pathways, and ultimately promote cell proliferation [63, 64, 65, 66, 67, 68]. In the present study, the detection of the expression of these proteins in the thiamine feeding experiment suggested the roles of IGFBP2/IGF1‐ERK and IGFBP2/IGF1‐PI3K/AKT in promoting ruminal cell proliferation and VFAs absorption, which in turn maintain the ruminal pH and prevent the occurrence of SARA.

Despite its contributions, this study has two primary limitations. First, the research focused exclusively on the regulatory mechanisms of SARA susceptibility and tolerance in dairy goats, and the key fungal metabolite thiamine was validated only in dairy goats. Second, the study was based on a relatively small sample size. It is anticipated that future research on the SARA, involving larger cohorts and a broader range of ruminant species, will build upon the theoretical foundation provided here.

## 4 Experimental Section

### 4.1 Animals, Experimental Design, and Sample Collection

#### 4.1.1 SARA‐ and SARA‐Tolerant Model Construction

Thirty‐six healthy multiparous dairy goats of similar body weight (40 ± 4 kg, 2–3 years) with rumen fistula were selected for the present study. Twelve dairy goats were randomly selected to feed a low‐concentrate diet at a 3:7 concentrate‐to‐forage ratio as control goats (CON, *n* = 12). The remaining 24 dairy goats were selected as the experimental goats by increasing the concentrate to‐ forage ratio to 7:3. The ingredients and nutritional components of the diets are shown in Table S1, Supporting information. One kilogram of dry matter from the TMR was fed daily at 08:00 and 17:00. There was a one‐week prefeeding period before the experiment. The 30% concentrate diet for the goats was maintained for 1 week during the prefeeding period, and the normal feeding period was 5 weeks.

On days 14, 21, 28, and 35 of the experiment, blood and rumen fluid samples were collected at 0, 2, 4, and 6 h after afternoon feeding. Blood was collected through the jugular vein into a heparin sodium tube as an anticoagulant and centrifuged at 3000× g for 20 min at 4°C to separate the plasma, which was subsequently stored at −80°C. Rumen fluid was collected from the rumen fistula, and 10 mL was used to immediately determine the pH; the remaining 40 mL was stored at ‐80°C. After real‐time pH detection on the 21st day of the trial, goats whose pH was less than 5.8 for more than 3 h in high‐concentrate feeding were selected as SARA‐susceptible goats (HCS, *n* = 8), and the other goats fed high‐concentrate diets were SARA‐tolerant goats (HCT, *n* = 16). The goats (including CON, HCS, and HCT groups) were further maintained for 2 weeks after SARA identification. Five dairy goats from each group were slaughtered on the last day of feeding (Figure 1A and Figure S1A, Supporting Information). The rumens of dairy goats were separated and weighed, and three rumen epithelial samples were collected from the rumen dorsal sac and abdominal sac and stored at −80°C.

#### 4.1.2 Thiamine Supplementation in High‐Concentrate Diets

Another fifteen healthy dairy goats (36 ± 2 kg) were randomly divided into CON (with 30% concentrate, *n* = 5), HCD and HCD‐T groups (with 70% concentrate, *n* = 5, separately) and fed different diets according to Table S2, Supporting Information, at 07:00 and 18:00 daily, with supernumerary 200 mg of thiamine/kg of dry matter to HCD‐T goats [60, 69, 70, 71]. The trial lasted for 12 weeks and 2 weeks for prefeeding with a 30% concentrate diet (Table S2, Supporting Information). On the last day of the trial period, the goats in each group were slaughtered at 11:00 am. One epithelial tissue sample from the rumen abdominal sac was collected and stored at −80°C.

### 4.2 Morphology of the Rumen Epithelial Papillae

The rumen epithelium was subjected to histological analysis via hematoxylin‒eosin (H and E) staining. Formaldehyde‐fixed epithelial papillae were dehydrated, sliced, and embedded in optimal cutting temperature compound. The sections were stained with hematoxylin and eosin before being photographed at 100× magnification in a Nikon ECLIPSE Ni‐U (Nikon Co., Ltd., Japan).

### 4.3 Determination of VFAs and LPS Concentrations in the Rumen Fluid and Blood

The rumen fluid was centrifuged at 13,000 × g for 10 min, the supernatant was obtained, and the concentrations of VFAs in the rumen fluid supernatant and plasma were measured as previously described [72]. One milliliter of metaphosphoric acid (250 g L^−1^) was added for protein precipitation, and 200 µL of crotonic acid (10 g L^−1^) was added as an internal standard. The VFAs were separated and quantified via an Agilent 7820 A GC system equipped with a polar capillary column (AE‐FFAP, dimensions: 30 m × 0.25 mm × 0.33 µm) and a flame ionization detector (FID). The method used for detecting LPS has been described previously [73]. Briefly, 100 µL of rumen fluid or plasma was added to a test tube and incubated with 100 µL of Limulus reagent and chromogenic substrate solution at 37°C. Then, the azotization reagent was added, the absorbance was read at 545 nm via a microplate reader (model 3550; Bio‐Rad, Hercules, CA), and the LPS concentration was calculated according to the standard curve.

### 4.4 Determination of Plasma Inflammatory Factors

The concentrations of IL‐1β, IL‐6, IL‐10, TNF‐α, and IL‐17 in the plasma of dairy goats were detected using the respective ELISA kits with the double antibody sandwich method, following the instructions provided by the manufacturer FANKEW. Specifically, prepared the reagents, samples, and standards, and dispensed them into the microplate. Incubated at 37°C for 30 min, and washed 5 times. HRP‐conjugate reagent was added, and the mixture was incubated at 37°C for 30 min and washed 5 times. Chromogenic solutions A and B were sequentially added, followed by an incubation period of 10 min at 37°C. Finally, the stop solution was added, and the optical density (O.D.) was measured.

### 4.5 Determination of Thiamine in the Rumen Fluid, Rumen Epithelium, and Plasma

The rumen epithelium was cut to 0.5 cm × 0.5 cm and homogenized via a high‐throughput tissue grinder (SCIENTZ‐48 L, China) at 70 Hz for 10 s 10 times. The rumen epithelium homogenates and ruminal fluids were subjected to centrifugation for 15 min at 3500 × g and 4°C. The relative concentrations of thiamine in the rumen epithelium, rumen fluid, and plasma were tested via the double‐antibody sandwich method using a Goat VB1 Elisa Kit (TSZ Biological Trade Co., Ltd., MA, USA). In accordance with the assay procedure, all standards were prepared, samples were added, biotinylated anti‐IgG and streptavidin‐HRP were added, and after incubation, the microwells were washed 4 times with liquid. Finally, chromogen solutions A and B were added, and after incubation for 15 min, the stop solution was added. The color of the liquids in the microwell changed from blue to yellow, and the O.D. at 450 nm was determined within 15 min.

### 4.6 Real‐Time Quantitative PCR and Western Blot

Rumen epithelial samples were ground and homogenized with lysis buffer for subsequent RNA and protein extraction. Total RNA was extracted via the Molpure Cell/Tissue Total RNA Kit (YEASEN, China) and quantified via a Nanodrop 2000 spectrophotometer. For mRNA analysis, 1 µL of total RNA was reverse transcribed with a PrimeScript RT Kit (Takara, Japan). For miRNA analysis, 1 µL of total RNA was converted to cDNA via a Mir‐X miRNA First‐Strand Synthesis Kit (Takara, Japan). Quantitative real‐time PCR was conducted on an Applied Biosystems QuantStudio 3, with the primer sequences listed in Table S3, Supporting Information. The differences in mRNA and miRNA expression levels were calculated via the 2^−ΔΔCT^ method.

The homogenate of the rumen epithelium was lysed in ice‐cold lysis buffer, and the protein concentration was determined via a BCA protein assay kit (Beyotime, China). The lysate and 5× loading buffer were combined with the protein supernatant for denaturation via heat treatment. For Western blot analysis, isolated protein samples were subjected to SDS‒PAGE via YoungPAGE Bis‒Tris gels before being transferred onto Immobilon‒PSQ PVDF membranes. The PVDF membrane was blocked with a solution of 5% nonfat milk powder diluted in TBST buffer. The samples were subsequently incubated overnight at 4°C with primary antibodies. After being washed with TBST, the membrane was incubated for two hours at room temperature with a goat anti‐rabbit IgG (H + L) HRP conjugate. Finally, the blots were visualized via a fluorescence imaging system (Tanon, China), and the grayscale values were calculated via ImageJ software.

### 4.7 Metagenomic Sequencing and Data Processing

The collected ruminal fluid was used to extract DNA by the E.Z.N.A. A soil DNA kit (Omega Biotek, US), and the concentration and purity of the DNA were examined with a YBS‐380 and NanoDrop2000. Agarose gel electrophoresis (1%) was used to detect DNA integrity, after which the DNA was fragmented, and approximately 400 bp fragments were selected. A pair‐end DNA library was constructed via NEXTFLEX Rapid DNA‐Seq (Bioo Scientific, US). After bridge PCR amplification, metagenomic sequencing was conducted via the Illumina NovaSeq/HiSeq Xten platform (Illumina, US).

The quality of the raw data was analyzed via fastp (https://github.com/OpenGene/fastp) and Trimmomatic [74]. The adapter at the 3’ end and 5’ end of the sequence were removed, and low‐quality reads that were shorter than 50 bp or whose base mass value was lower than 20 were cut off. The host reads were removed by mapping to the Capra hircus reference genome in BWA (v0.7.17) to obtain clean reads of the microbiome. Megahit [75](https://github.com/voutcn/megahit) was used to carry out kmer iteration to assemble short contigs in the succinct de Bruijn graph. The open reading frame (ORF) prediction of each assembled contig was performed via Prodigal. ORFs with nucleic acid lengths greater than or equal to 100 bp were selected and translated into amino acid sequences. CD‐HIT (http://www.bioinformatics.org/cd‐hit/) was used to construct a nonredundant gene catalogue with 90% identity and 90% coverage. High‐quality reads were aligned to the nonredundant gene catalogue to obtain the read abundance in every sample via SOAPaligner (http://soap.genomics.org.cn/) with 95% identity. Trans per million (TPM) was used to normalize the gene abundance of different samples to avoid deviation of sequencing.

BLASTP (BLAST Version 2.2.28+, http://blast.ncbi.nlm.nih.gov/Blast.cgi) was employed to compare the nonredundant gene set with the NR database via a BLAST alignment parameter set to an expected e‐value of 1e‐5. The species annotation results were subsequently obtained from the corresponding taxonomic information database of the NR library. The abundance of each species is then calculated by summing the corresponding gene abundances. To facilitate Kyoto Encyclopedia of Genes and Genomes (KEGG) annotations, Diamond software was used to query against the KEGG database (http://www.genome.jp/keeg/), employing an E value threshold of 1e‐5. Similarly, for annotating carbohydrate‐active enzymes, hmmscan (accessible via http://HMMER.janelia.org/search/hmmscan) was employed with an E‐value cut‐off of 1e‐5 to probe the carbohydrate‐active enzymes (CAZy) database (http://www.cazy.org/).

### 4.8 Rumen Epithelium Single‐Nucleus RNA Sequencing and Data Processing

The single‐nucleus suspension was prepared by extracting nuclei from frozen rumen epithelial samples as previously described [76]. A final concentration of 1000 nuclei per microliter was used for loading on a 10× Chromium. A single nucleus was isolated by association with beads loaded with a unique molecular identifier (UMI) in a gel beads‐in‐emulsion (GEM). The GEM was exposed to cell lysis buffer, and the polyadenylated RNA molecules were hybridized onto microbeads and isolated in a single test tube for reverse transcription. During cDNA synthesis, each cDNA molecule was tagged on the 5’ end (i.e., the 3’ end of a messenger RNA transcript) with both the UMI and the cell label, indicating its cell of origin, and cDNA amplification and quality checks were performed. The qualified cDNA was subsequently subjected to next‐generation sequencing of the library. The sequencing libraries were quantified via a high‐sensitivity DNA chip (Agilent) on a Bioanalyzer 2100 and a Qubit high‐sensitivity DNA assay (Thermo Fisher Scientific, New York, USA). The libraries were subsequently sequenced on an Illumina NovaSeq Xplus platform (Illumina, San Diego, USA) in PE150 sequencing mode.

The quality of the raw read data was analyzed via Fastp (https://github.com/OpenGene/fastp). The Cell Ranger (v7.1.0) pipeline was used to process the reads, and the reads generated from the Illumina sequencing output were subsequently aligned to the Capra hircus reference genome in STAR [77] to obtain quality control results such as high‐quality cell counts and genome comparison information from the sample data. The gene‒barcode matrix was subsequently imported into the Seurat (v4.1.1) R toolkit for quality control and further analysis of our single‐cell RNAseq data [78]. All the functions were executed with default parameters. We subsequently visualized the clusters on a 2D map created via t‐distributed stochastic neighbor embedding (t‐SNE) [79]. Through singleR (http://www.bioconductor.org/packages/devel/bioc/html/SingleR.html) analysis [80], we determined the cell type of each cell population needed to recognize the main types of rumen epithelium. The epithelial cell subclusters were subdivided according to known marker genes according to the bovine single‐cell database of Zhejiang University (http://cattlecelllandscape.zju.edu.cn). The custom gene set was scored via the AddModuleScore function [81].

### 4.9 Rumen Epithelium Transcriptome Sequencing and Data Processing

Transcriptome sequencing was performed on the rumen epithelium of 15 dairy goats after slaughter. First, total RNA was extracted from the tissue using TRIzol reagent (Thermo Fisher, New York, USA). A NanoDrop2000 (Thermo Fisher Scientific, USA) was used to determine the concentration and purity of the extracted RNA, and agarose gel electrophoresis was used to determine RNA integrity. An Agilent 5300 (Agilent, USA) was used to determine the RNA integrity number (RIN), and to ensure that the total RNA concentration was not less than 1 µg, the RIN was greater than 6.5, and the concentration was greater than 30 ng/µL. Magnetic beads with oligo(dT) were used for A‐T base pairing with polyA to enrich total RNA. The mRNA was randomly broken into small fragments of approximately 300 bp by the addition of the fragmentation buffer. Under the action of reverse transcriptase, six‐base random hexamers were added to invert the mRNA template to synthesize strand cDNA, after which double‐strand synthesis was carried out to form a stable double‐strand structure. End Repair Mix was added to make the sticky end of the cDNA flat, and then an “A” base was added to the 3' end to connect the Y‐shaped splice. Target cDNA fragments 300 bp in length were selected and purified via 2% agarose (Biowest, Spain). PCR amplification was subsequently performed via Phusion DNA polymerase (NEB, USA) for 15 cycles. After quantitative detection, the Illumina NovaSeq 6000 platform (Illumina, San Diego, USA) was used for sequencing.

Quality control of the sequencing data was performed via fastp [82]. HISAT2 was subsequently used to compare the clean reads after quality control with the reference genome of Capra hircus, with default parameters [83]. The expression levels of genes and transcripts were quantitatively analyzed via RSEM, with TPM serving as the quantitative index [84]. Intersample Venn, correlation, and PCA analyzes were performed on the basis of the expression matrix. In accordance with the results of the quantitative detection of gene expression, DESeq2 was used to analyze the DEGs among the samples. DEGs with a |log2FC|>1 and an FDR value < 0.05 were considered to be significant DEGs. KOBAS (http://kobas.cbi.pku.edu.cn/home.Do) was used for KEGG pathway enrichment analysis [85].

### 4.10 Rumen Epithelium Small RNA Transcriptome Sequencing and Data Processing

The process of RNA extraction was the same as above. One microgram of total RNA was used as the input material for small RNA library preparation. A QIAseq miRNA Library Kit (Qiagen) was used to generate the sequencing libraries. The activated 5' and 3' adaptors were then individually ligated to the RNA molecules. The adaptor‐ligated RNA was transcribed into first‐strand cDNA via reverse transcriptase and random primers. PCR was performed for 11–12 cycles to amplify fragments of appropriate size. These fragments were subsequently separated via a 6% Novex TBE PAGE gel. Following quantification via Qubit4.0, the single‐end RNA‐seq libraries were sequenced on an Illumina NovaSeq X Plus sequencer.

The raw data were first processed for quality control through fastp [82]. After this stage, clean reads were obtained by removing adapter sequences from the 3' end and reads containing poly‐N and low‐quality bases (anger base quality of <20) at the 3' end. The sequences with sizes ranging from 18 to 32 nt were screened, and the chromosomal locations aligning with the Capra hircus reference genome were annotated via Bowtie [86]. The mapped small RNA tags were first used to identify known miRNAs by alignment with the miRBase V22 database (http://www.mirbase.org/) as a reference. The unannotated tags were subsequently predicted and identified as novel miRNAs via miRDeep2 software [87] on the basis of their tag positions in the genome and hairpin structures. The expression level of each miRNA was calculated according to the transcripts per million reads (TPM) method. Differential expression analysis was performed via DESeq2 [88]. miRNAs with a FC ≥ 1.5 or FC ≤ 2/3 and FDR < 0.05 were considered to be significantly differentially expressed miRNAs. Target gene predictions of the miRNAs were performed with miRanda. Spearman correlation analysis was performed to screen for miRNA–mRNA pairs that exercised their functions through posttranscriptional regulation of their target genes.

### 4.11 Bioinformatics and Statistical Analysis

The LPS and VFAs contents of the rumen and plasma, as well as the mRNA expression measured by qPCR, were statistically evaluated via one‐way ANOVA, and the pH of the ruminal fluid was statistically evaluated via repeated measures in a general linear model via SPSS Statistics 27 (Armonk, USA). The α diversity and beta diversity of the metagenomic data were analyzed in R‐studio with the vegan package. PCoA was executed via the Bray‒Curti's distance algorithm, and the Adonis method was employed to assess the disparities in species composition across various groups, with 999 permutation tests in the *R* package pairwiseAdonis. To analyze the species and functional disparities between any two groups, LEfSe analysis was conducted with a nonparametric factorial Kruskal‒Wallis sum‒rank test, the Wilcoxon rank‒sum test and linear discriminant analysis with the R packages tidyverse/magirittr, phyloseq and microbiomeMarker, and LinkET was used to conduct the Mantel test. The genome of *Aspergillus bombycis* was downloaded from NCBI RefSeq assembly GCF_001792695 [89]. The protein sequence was aligned with KEGG database by GhostKOALA (https://www.kegg.jp/ghostkoala/). Spearman's correlation coefficient was used in all monotonicity correlation analyzes in this study, and functional enrichment analysis was based on KOBAS (http://kobas.cbi.pku.edu.cn/), with an FDR *p* value < 0.05. The data were visualized with R‐studio (version 4.3.2) with the default parameters of the R packages ggalluvial and ggplot2. Cytoscape (v3.9.0) was used to plot the correlation network.

## Author Contributions

SRW, XDC, JHY and JYX conception and design the research; XDC, YM, JRR, GHX, PLZ, WHJ, QQD, YQX, JHY, SRW, JYX were response for the animal feeding and sample collection; SRW, XDC, JYX, YM, and JHY were response for development of methodology; SRW, JHY, JYX, XDC, and YM were response for the acquisition of data, SRW, JHY, JYX were response for analysis and interpretation of data; SRW, JYX and JHY were response for writing, review, and/or revision of the manuscript. All authors read, edited, and approved the final manuscript.

## Ethics Statement

The experiment was conducted at the Animal Research and Technology Center of Northwest A&F University (Yangling, Shaanxi, China). All the experimental designs and protocols used in the present study were approved by the Institutional Animal Care and Use Committee (IACUC) of Northwest A&F University (Shaanxi, China; approval number: NWAFU‐DK‐2022155).

## Conflicts of Interest

The authors declare no conflicts of interest.

## Supporting information




**Supplementary File 1**: exp270142‐sup‐0001‐SuppMat.zip.

## Data Availability

All the raw sequencing data generated in this study have been submitted to the NCBI BioProject database (https://www.ncbi.nlm.nih.gov/bioproject/) under accession numbers PRJNA1165831 and PRJNA1165347. Other data generated or analyzed for this study are included in this paper and the supplementary material.
