# Supplementary material for: Rumen Mycobiome Thiamine Metabolism Contributes to Subacute Rumen Acidosis Tolerance in Goats Through Enhancing Epithelial Cell Proliferation via IGFBP2/IGF1 Axis Activation
Source: Exploration (Beijing). 2026 Feb 24;6(2):70142. doi: 10.1002/exp2.70142 (PMC13094527; doi:10.1002/exp2.70142)
Supplement: Supplementary file 1 — exp270142‐sup‐0001‐SuppMat.zip. [file EXP2-6-70142-s001.zip › Supplemental figures legend.pdf]

Figure S1 SARA and rumen epithelial inflammation occurred in HCS goats. (A) Dynamic changes in rumen pH 1–6 hours after feeding in the afternoon among CON, HCS, and HCT dairy goats (n = 5, separately). (B) Upset map showing the gene numbers of each group, including their common and particular genes, in CON, HCS and HCT dairy goats (n = 5, separately). (C) Differential expression of genes upregulated in the HCS group that participate in the IL-17 signalling pathway, the TNF signalling pathway and the Toll-like receptor signalling pathway among CON, HCS and HCT dairy goats (n = 5, separately). The pH values (A) are expressed as the means ± SEMs, and the repeated-measures general linear model for pH was analysed, followed by the LSD and DUNCAN tests. $*P < 0.05$, $**P < 0.01$, $***P < 0.001$ indicate significance.

Figure S2 Rumen epithelial morphology of HCT dairy goats was normal, and genes related to cell proliferation were upregulated. (A) Differences in the expression of *WNT5B*, *HMOX1* and *NQO1* between HCT and CON goats (n = 5, separately). (B) Concentrations of volatile fatty acids in the rumen fluid 2 hours after feeding, specifically, the concentrations of total volatile fatty acids, acetate, propionate, butyrate, and valerate and the ratio of acetate to propionate (n = 5, separately). (C-D) Differences in the number of papillae (C) and papillae width (D) among CON, HCS and HCT dairy goats after slaughtering (n = 5, separately). (E) HE-stained sections of rumen epithelial papillae from CON, HCS, and HCT dairy goats. The statistical method is the same as that in Figure S1.

Figure S3 The top three marker genes of each of the 26 clusters.

Figure S4 Comparison of the number of various types of cells in the rumen epithelium of dairy goats in the CON, HCS and HCT groups identified by single-nucleus RNA sequencing and

differences in epithelial cell subclusters. (A) The differences in t-SNE maps among 10 subclusters identified in epithelial cells among CON HCS and HCT dairy goats (n = 1, separately). (B) Demonstration of several marker genes in basal cells, granule cells and spinous cells. (C) The t-SNE map showed the gene set scores of genes upregulated in HCT dairy goats compared with those in CON (above) and HCS (below) dairy goats in subclusters of epithelial cells (n = 1, separately).

Figure S5 Differences in microbiome richness and diversity among CON, HCS and HCT dairy goats. (A-B) α diversity of all the microbiomes (A) and fungi (B) identified in the rumens of CON, HCS and HCT dairy goats, including all the metrics, such as the Shannon, Simpson, Ace and Chao indices (n = 5, separately). (C) β diversity of all the microbiomes identified in the rumens of CON, HCS and HCT dairy goats via PCoA and a permutation test 999 times to determine the statistical significance (n = 5, separately).

Fig S6 LEfSe analysis to screen the significantly differential KEGG pathways at level 3. (A-B) The significantly differential KEGG pathways at level 3 between HCS and CON (A) and between HCT and HCS (B), with LDA > 2 and a P value < 0.05 (n = 5, separately).

Figure S7 Differences in KOs between HCT and CON dairy goats and correlation analysis between the contents of thiamine and fungi significantly related to thiamine metabolism and *NFS1*. (A) LEfSe analysis to screen the different species between HCT and CON with LDA > 2 and a P value < 0.05 (n = 5, separately). (B-C) Spearman correlation analysis between the content of thiamine in plasma and the *Podospora comata* relative abundance (B), and the *Malassezia sympodialis* relative abundance (C) (n = 5, separately).

Figure S8 The target genes of differentially expressed miRNAs between HCT and CON and

between HCT and HCS were subjected to KEGG enrichment analysis, and the networks of correlations of KEGG pathways, miRNAs and genes were shown. (A) The differential miRNAs in the rumen epithelium between CON- and HCT-targeted genes were subjected to KEGG enrichment analysis (n = 5, separately). (B) The differential miRNAs in the rumen epithelium between the HCS- and HCT-targeted genes were subjected to KEGG enrichment analysis (n = 5, separately).

Figure S9 Multiomics analysis revealed that the key genes involved in thiamine metabolism in fungi inhibited miRNA expression to upregulate gene expression to affect cell proliferation and VFAs absorption. (A) Target relationship between chi-miR-17-26080 and *FAM3D*, as well as the comparison of relative expression of *FAM3D* between CON and HCT (n = 5, separately). (B) Target relationships between chi-miR-433 and its target genes *LEP* and *SGK1*.

Figure S10 The relative expression of *IGF1* mRNA (A) and the relative expression of the PI3K protein (B) (n = 5, separately).

Table S1 Ingredients and nutrient composition of the two diets.

Table S2 Ingredients and nutrient compositions of dairy goats fed 3 different types of diets.

Table S3 Primers and base sequences for real-time quantitative PCR.

Table S4 The KEGG enrichment analysis of significantly downregulated DEGs in the HCS and HCT groups.

Table S5 Cell type and number in each cluster.

Table S6 The number and proportion of different cell types in each sample.

Table S7 Maker genes of different types of epithelial cells.

Table S8 The number and proportion of different cell types of epithelial cells in each sample.

Table S9 The species and taxonomy of rumen fungi identified via the NR database.

Table S10 Total accounting of the microbiome in the rumen, including bacteria, fungi, archaea and viruses.

Table S11 The participating pathways and abundance of differential KO between HCT and CON dairy goats counted in TPM.

Table S12 The genes of *Aspergillus bombycis* annotated in the KEGG database.
