# Supplementary material for: Rumen Mycobiome Thiamine Metabolism Contributes to Subacute Rumen Acidosis Tolerance in Goats Through Enhancing Epithelial Cell Proliferation via IGFBP2/IGF1 Axis Activation
Source: Exploration (Beijing). 2026 Feb 24;6(2):70142. doi: 10.1002/exp2.70142 (PMC13094527; doi:10.1002/exp2.70142)
Supplement: Supplementary file 1 — exp270142‐sup‐0001‐SuppMat.zip. [file EXP2-6-70142-s001.zip › Supplemental_Fig_S4.pdf]

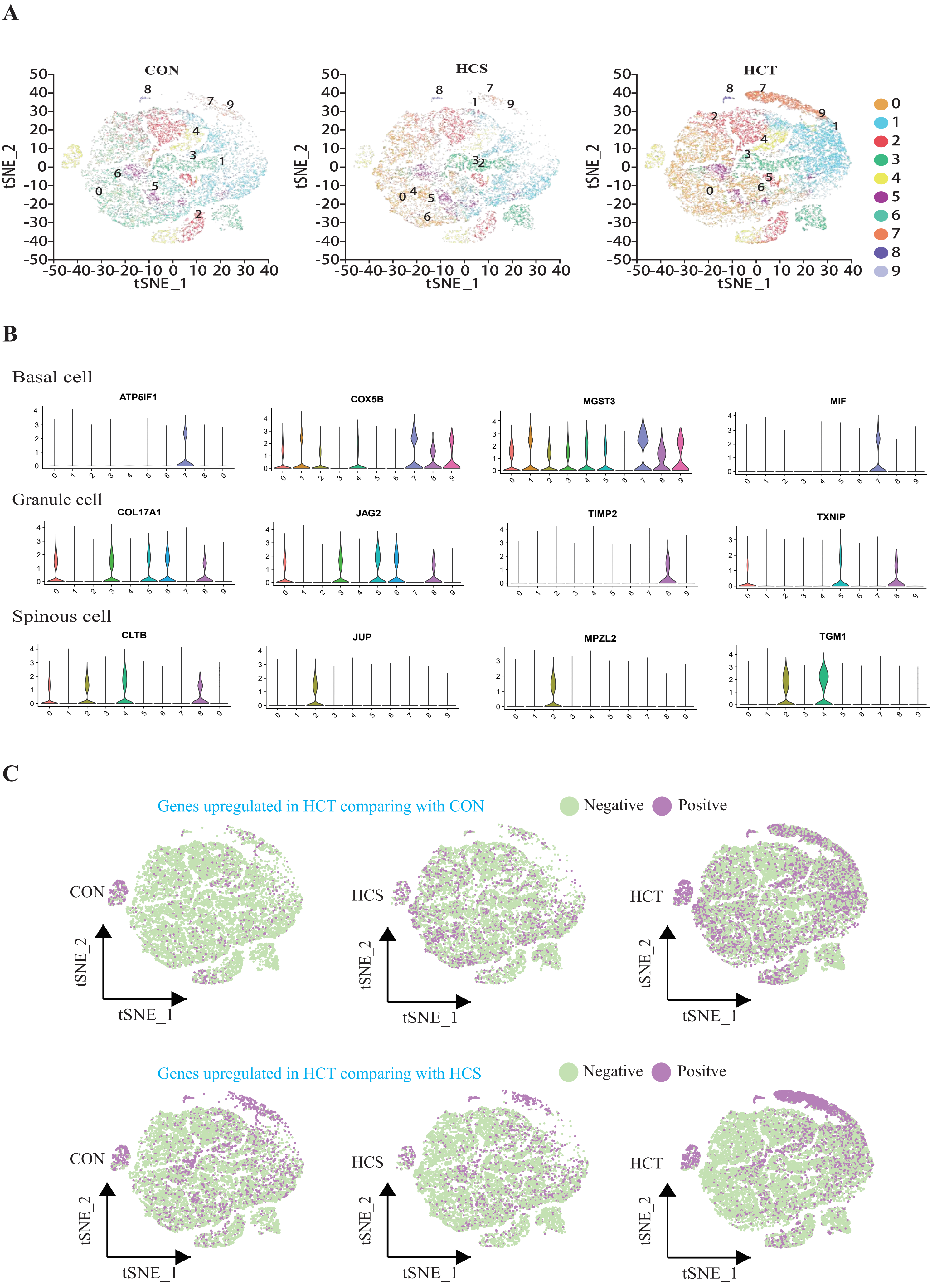
A
CON
HCS
HCT
tSNE_1
tSNE_2
50
40
30
20
10
0
-10
-20
-30
-40
-50
-50-40-30-20-10 0 10 20 30 40
0
1
2
3
4
5
6
7
8
9
B
Basal cell
ATP5IF1
COX5B
MGST3
MIF
Granule cell
COL17A1
JAG2
TIMP2
TXNIP
Spinous cell
CLTB
JUP
MPZL2
TGM1
C
Genes upregulated in HCT comparing with CON
Negative
Positve
CON
HCS
HCT
tSNE_1
tSNE_2
Genes upregulated in HCT comparing with HCS
Negative
Positve
CON
HCS
HCT
tSNE_1
tSNE_2
