# Supplementary material for: Rumen Mycobiome Thiamine Metabolism Contributes to Subacute Rumen Acidosis Tolerance in Goats Through Enhancing Epithelial Cell Proliferation via IGFBP2/IGF1 Axis Activation
Source: Exploration (Beijing). 2026 Feb 24;6(2):70142. doi: 10.1002/exp2.70142 (PMC13094527; doi:10.1002/exp2.70142)
Supplement: Supplementary file 1 — exp270142‐sup‐0001‐SuppMat.zip. [file EXP2-6-70142-s001.zip › Supplemental_Fig_S9.pdf]

A

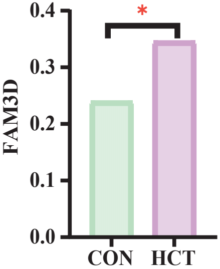

chi-miR-326-5p:
3' ucGGGUUGUCUGUCUGCUCc 5'
FAM3D 3’UTR:
5' caCCCTCTAGCCAGACGAGc 3'

B

chi-miR-433:
3' uguGGCUCCUCGGGUAGUACUa 5'
LEP 3’UTR:
5' aatCCAGGGAG - - CATCATGAa 3'

chi-miR-433:
3' uguGGCUCCUCGGGUAGUACUa 5'
SGK1 3’UTR:
5' actCAGAGGAGCTCATCATGGa 3'
