# Supplementary figures and images for: Rumen Mycobiome Thiamine Metabolism Contributes to Subacute Rumen Acidosis Tolerance in Goats Through Enhancing Epithelial Cell Proliferation via IGFBP2/IGF1 Axis Activation
Source: Exploration (Beijing). 2026 Feb 24;6(2):70142. doi: 10.1002/exp2.70142 (PMC13094527; doi:10.1002/exp2.70142)

**A**

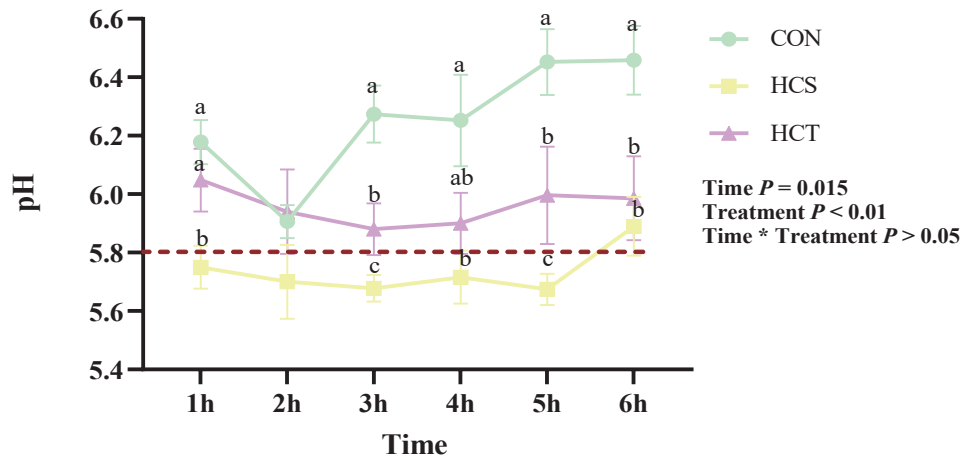

**B**

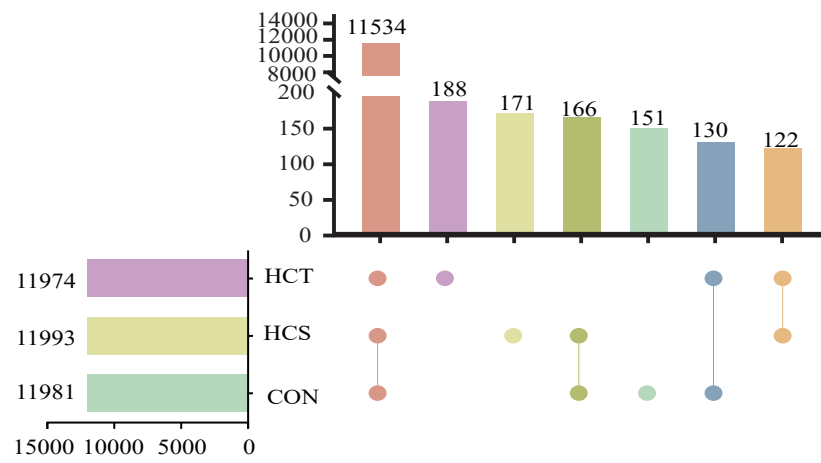

**C**

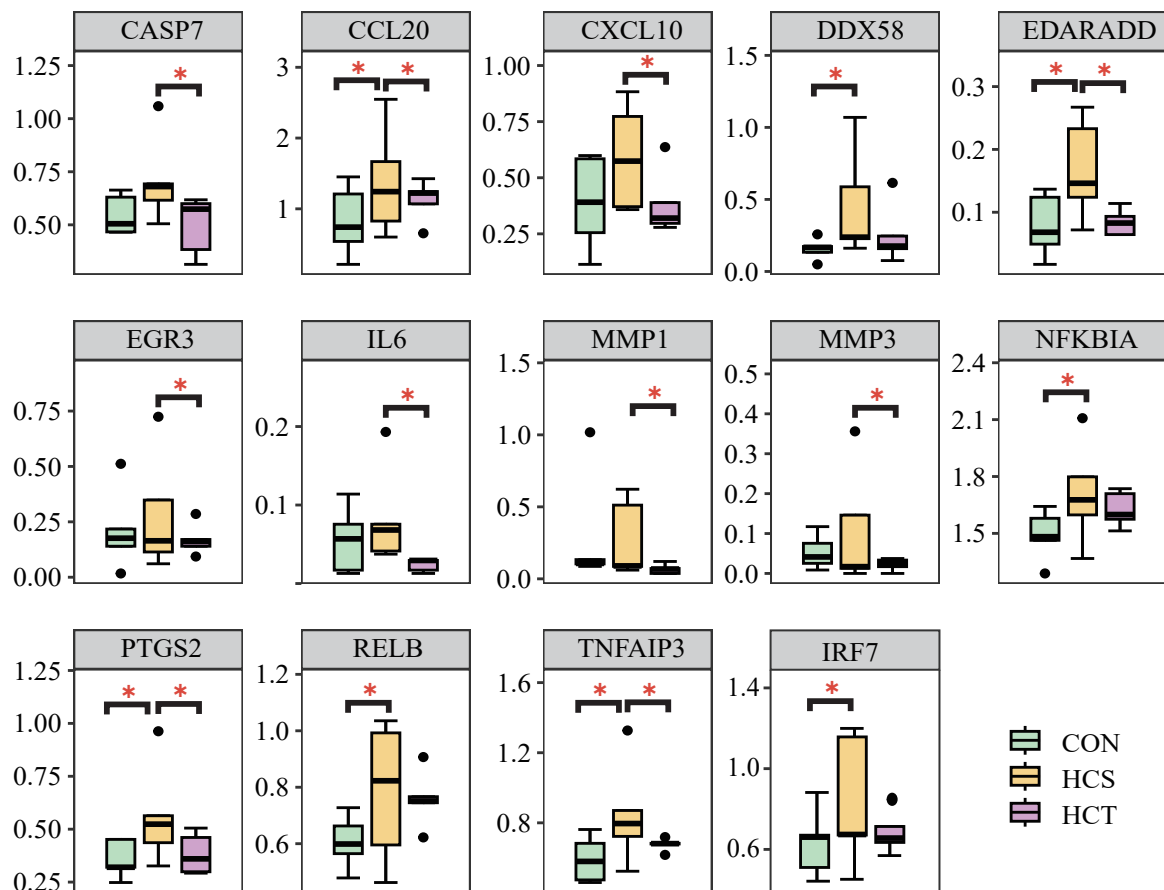

Supplement: Supplementary file 1 — exp270142‐sup‐0001‐SuppMat.zip. [file EXP2-6-70142-s001.zip › Supplemental_Fig_S1.pdf]

**A**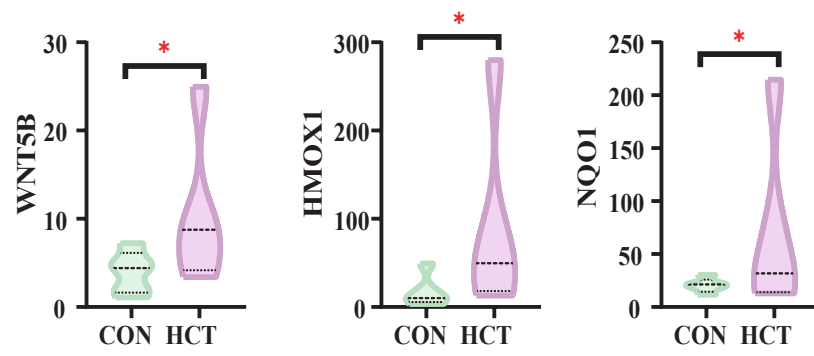**B**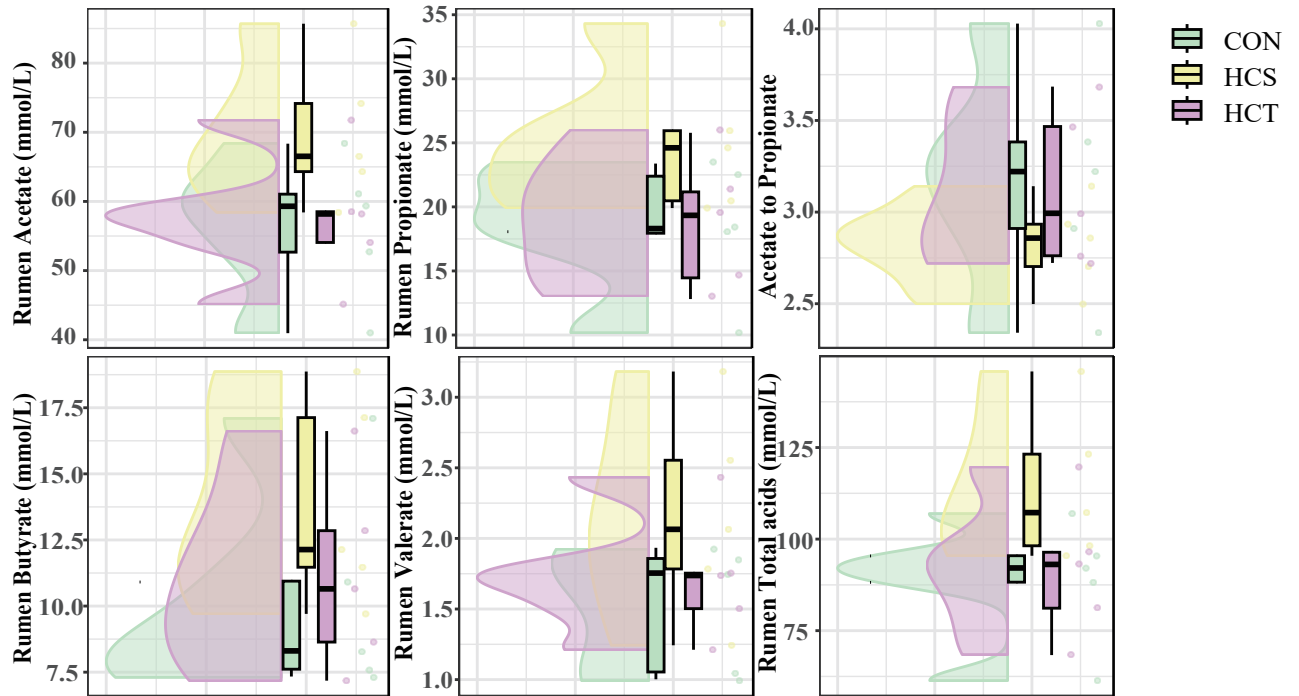**C**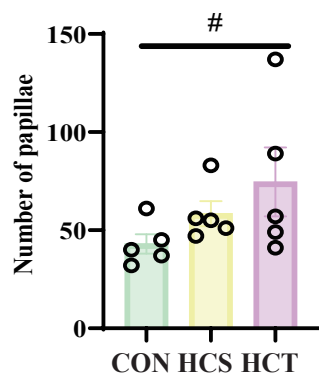**D**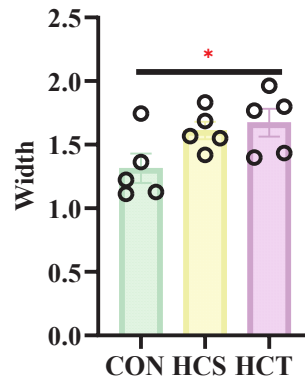**E**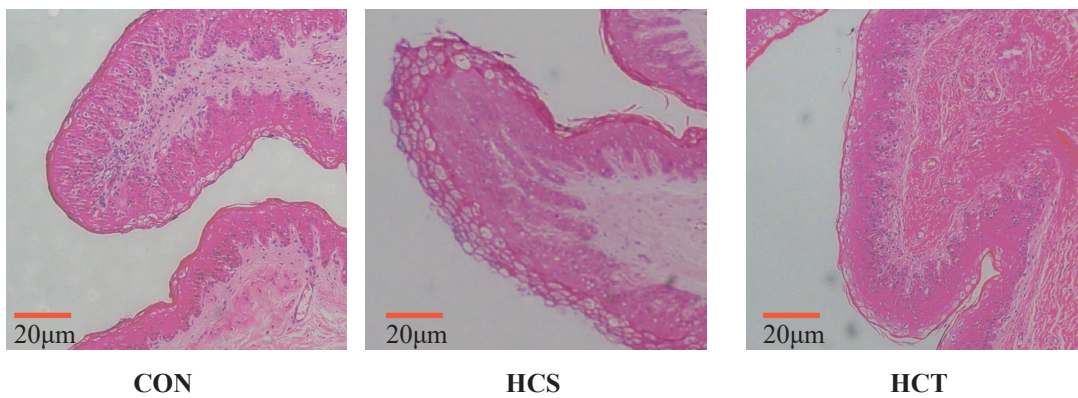

Supplement: Supplementary file 1 — exp270142‐sup‐0001‐SuppMat.zip. [file EXP2-6-70142-s001.zip › Supplemental_Fig_S2.pdf]

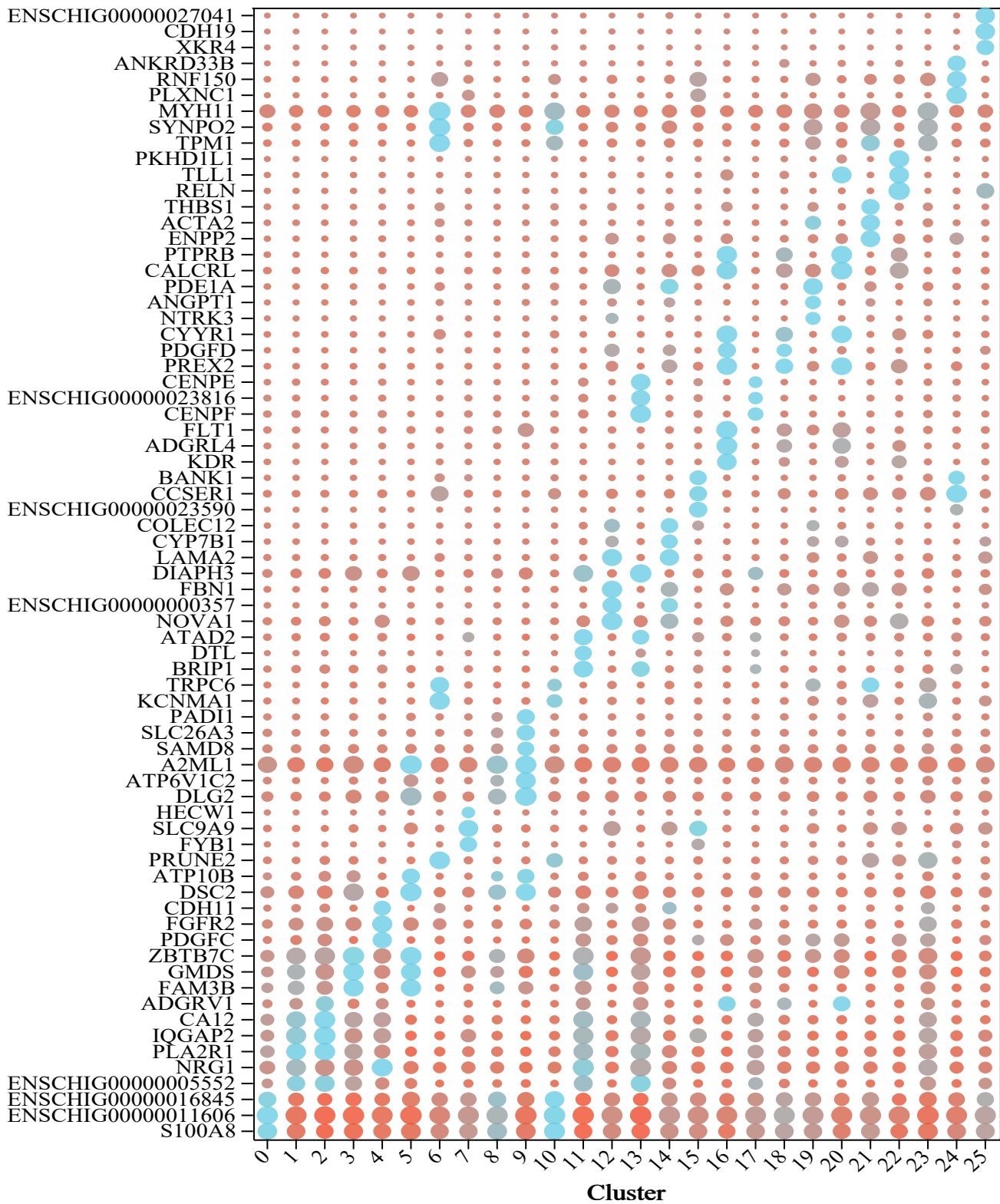

Supplement: Supplementary file 1 — exp270142‐sup‐0001‐SuppMat.zip. [file EXP2-6-70142-s001.zip › Supplemental_Fig_S3.pdf]

A

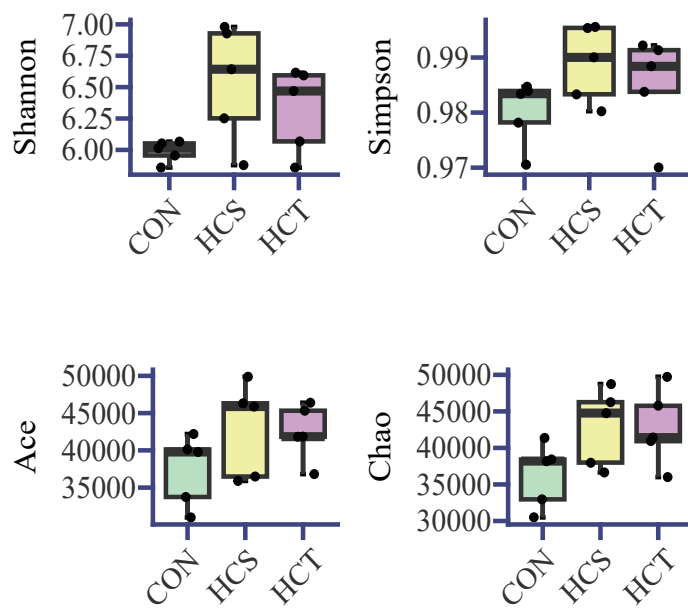

B

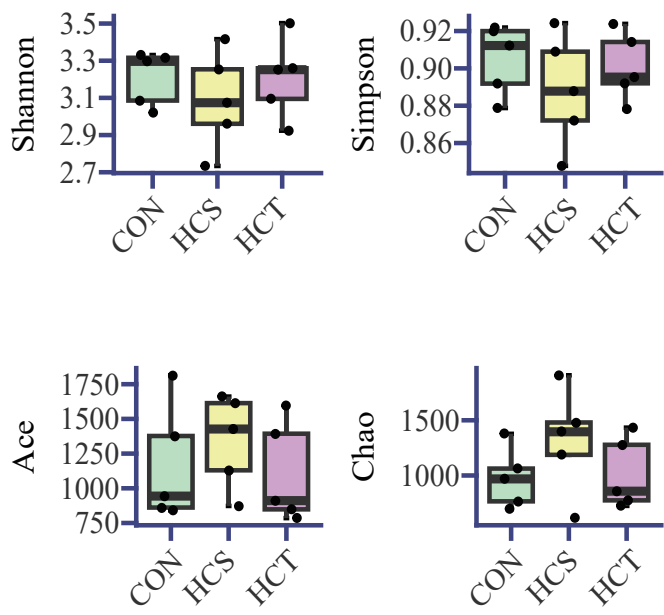

C

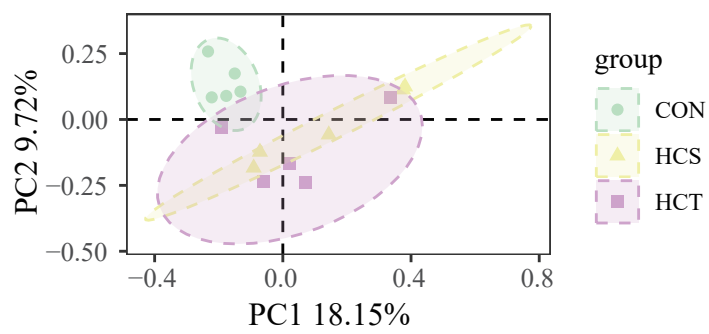

| pairs      | <i>P</i> value |
|------------|----------------|
| CON vs HCS | 0.013          |
| CON vs HCT | 0.009          |
| HCS vs HCT | 0.371          |

Supplement: Supplementary file 1 — exp270142‐sup‐0001‐SuppMat.zip. [file EXP2-6-70142-s001.zip › Supplemental_Fig_S5.pdf]

**A**

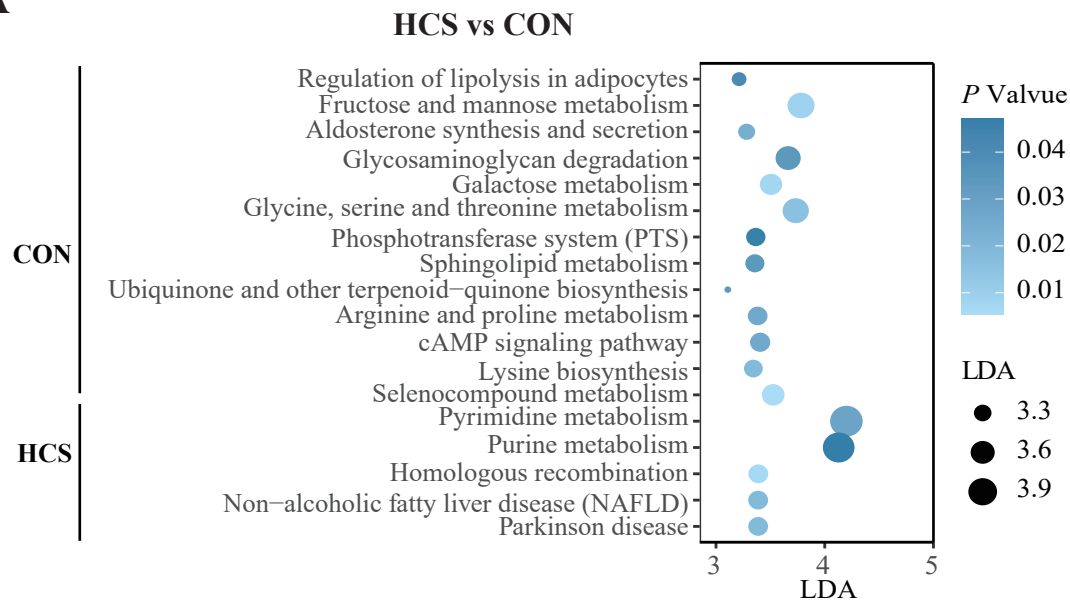

**B**

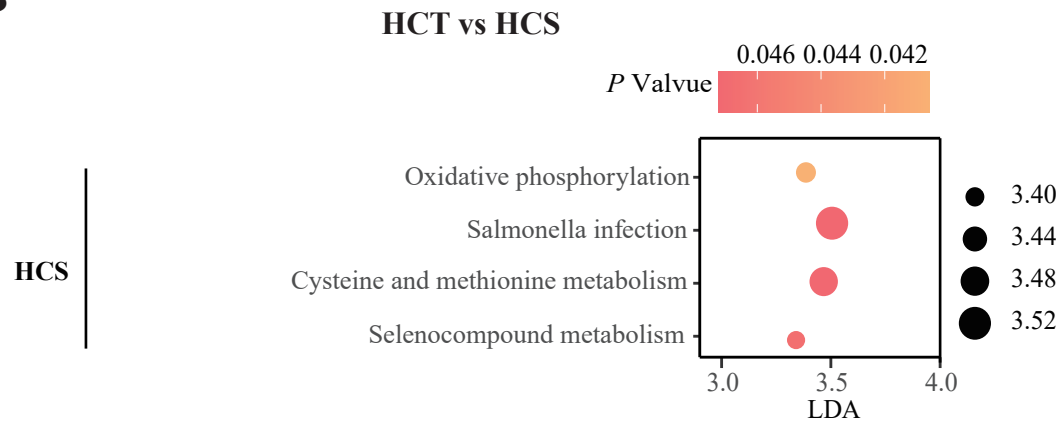

Supplement: Supplementary file 1 — exp270142‐sup‐0001‐SuppMat.zip. [file EXP2-6-70142-s001.zip › Supplemental_Fig_S6.pdf]

**A**

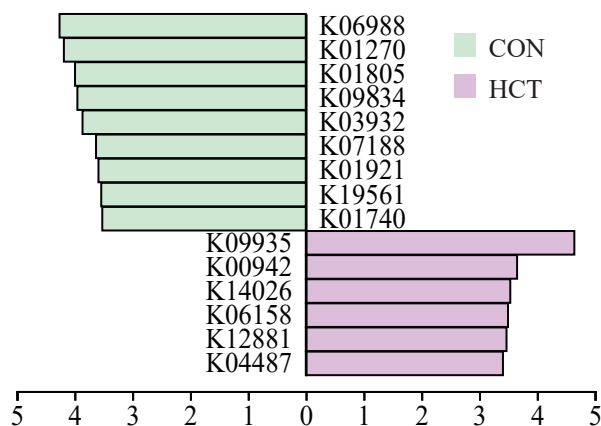

**B**

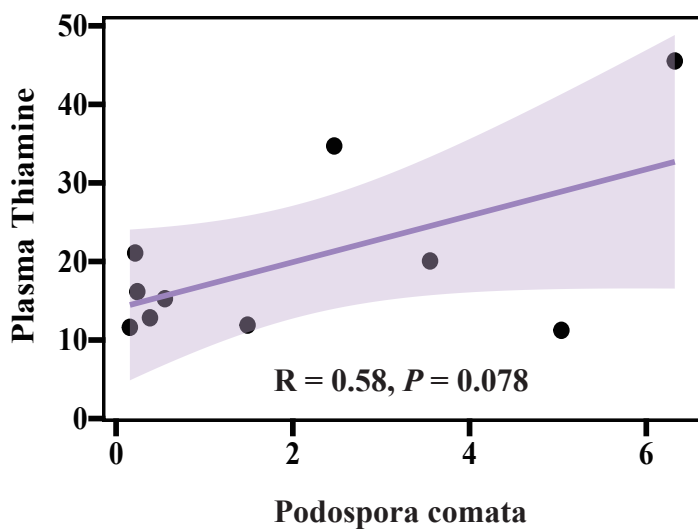

**C**

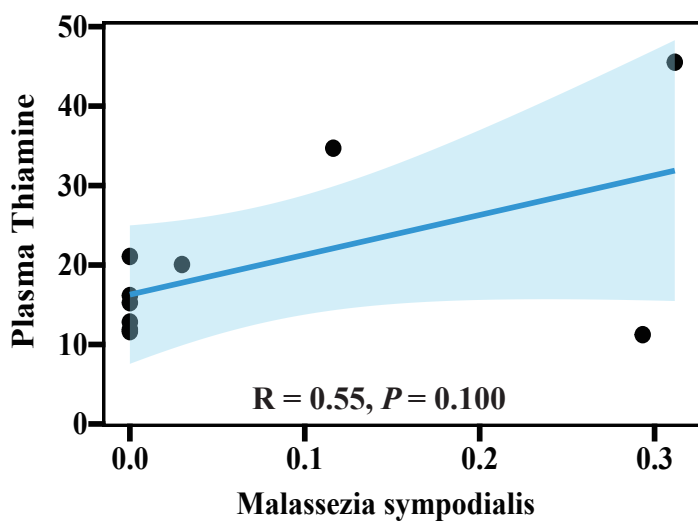

Supplement: Supplementary file 1 — exp270142‐sup‐0001‐SuppMat.zip. [file EXP2-6-70142-s001.zip › Supplemental_Fig_S7.pdf]

A

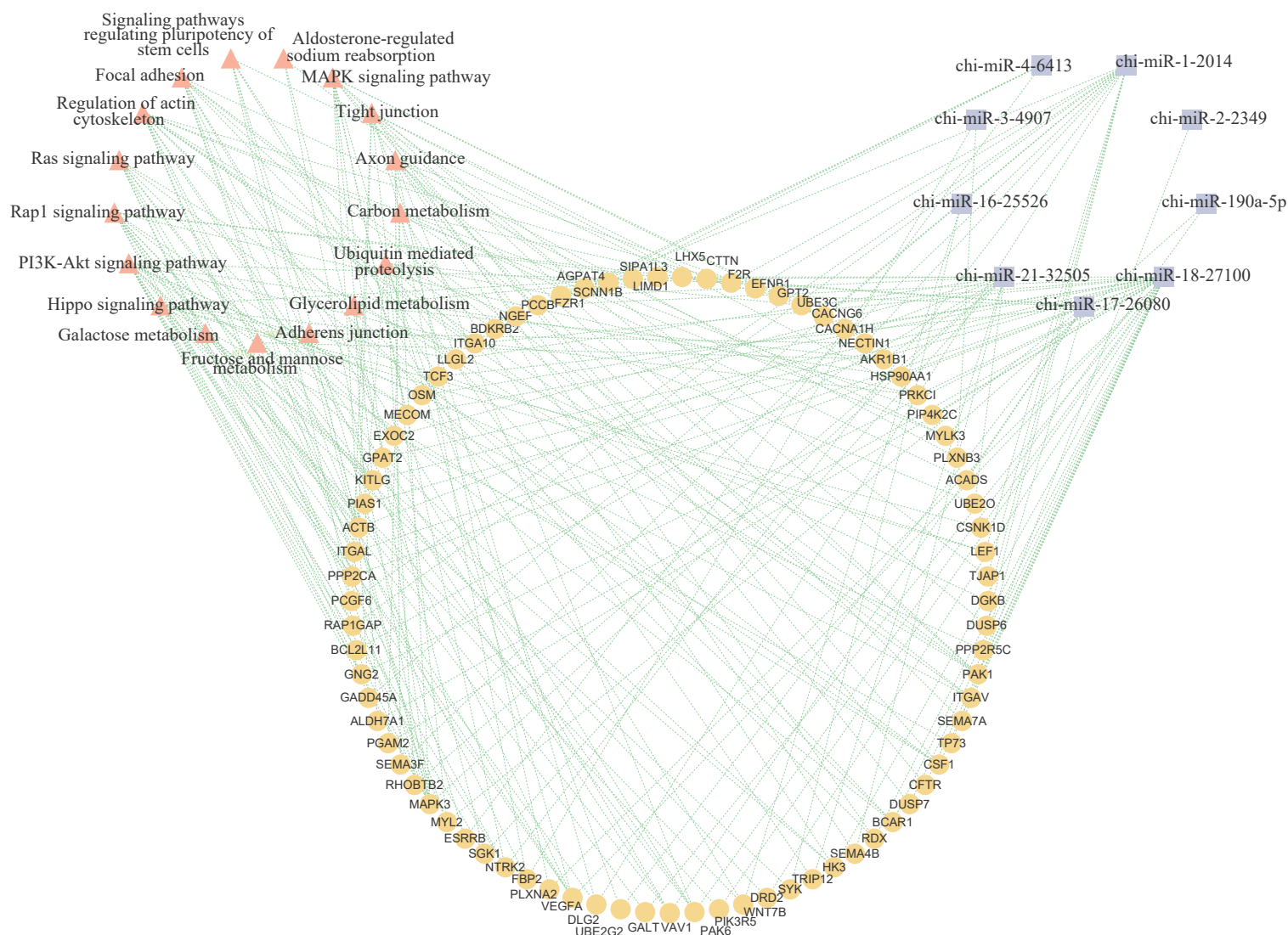

B

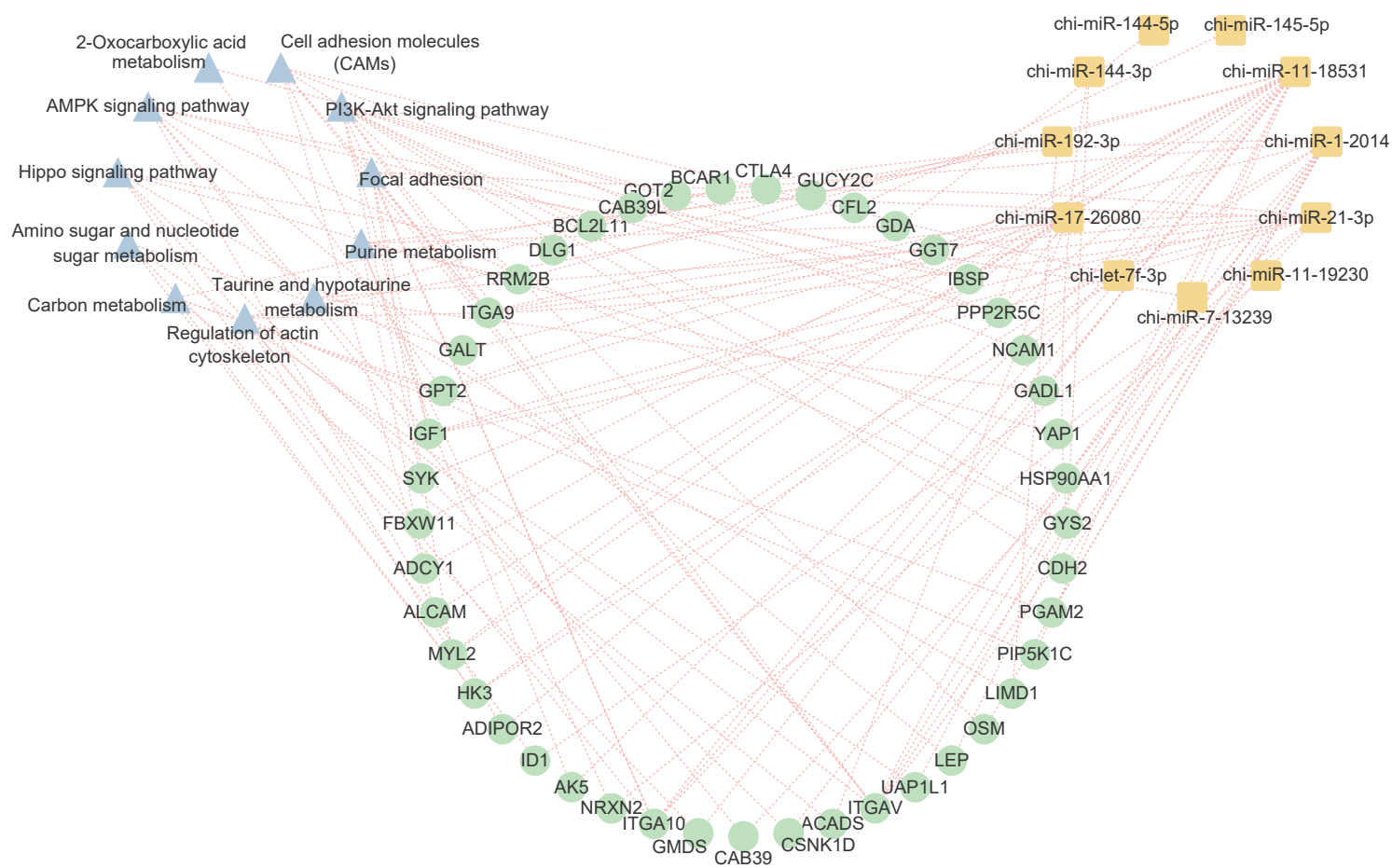

Supplement: Supplementary file 1 — exp270142‐sup‐0001‐SuppMat.zip. [file EXP2-6-70142-s001.zip › Supplemental_Fig_S8.pdf]

**A**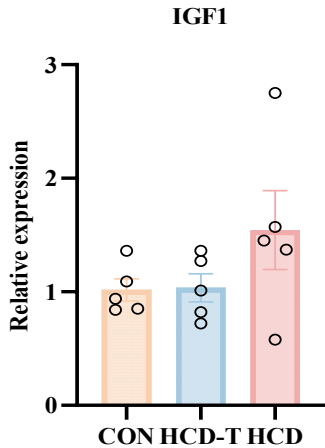**B**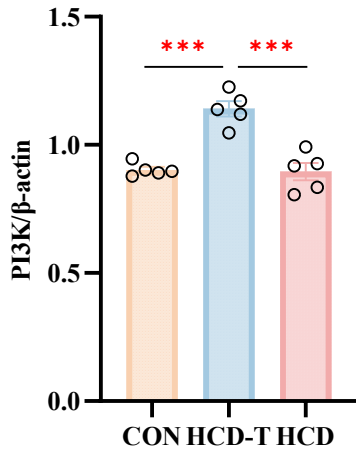

Supplement: Supplementary file 1 — exp270142‐sup‐0001‐SuppMat.zip. [file EXP2-6-70142-s001.zip › Supplemental_Fig_S10.pdf]
